# Characterization of genetic variants of *GIPR* reveals a contribution of β-arrestin to metabolic phenotypes

**DOI:** 10.1038/s42255-024-01061-4

**Published:** 2024-06-13

**Authors:** Hüsün S. Kizilkaya, Kimmie V. Sørensen, Jakob S. Madsen, Peter Lindquist, Jonathan D. Douros, Jette Bork-Jensen, Alessandro Berghella, Peter A. Gerlach, Lærke S. Gasbjerg, Jacek Mokrosiński, Stephanie A. Mowery, Patrick J. Knerr, Brian Finan, Jonathan E. Campbell, David A. D’Alessio, Diego Perez-Tilve, Felix Faas, Signe Mathiasen, Jørgen Rungby, Henrik T. Sørensen, Allan Vaag, Jens S. Nielsen, Jens-Christian Holm, Jeannet Lauenborg, Peter Damm, Oluf Pedersen, Allan Linneberg, Bolette Hartmann, Jens J. Holst, Torben Hansen, Shane C. Wright, Volker M. Lauschke, Niels Grarup, Alexander S. Hauser, Mette M. Rosenkilde

**Affiliations:** 1 Department of Biomedical Sciences, Faculty of Health and Medical Sciences, University of Copenhagen, Copenhagen, Denmark; 2 Novo Nordisk Foundation Center for Basic Metabolic Research, Faculty of Health and Medical Sciences, University of Copenhagen, Copenhagen, Denmark; 3 Department of Drug Design and Pharmacology, University of Copenhagen, Copenhagen, Denmark; 4 Novo Nordisk Research Center Indianapolis, Indianapolis, IN USA; 5 Indiana Biosciences Research Institute Indianapolis, Indianapolis, IN USA; 6 Department of Bioscience and Agro-Food and Environmental Technology, University of Teramo, Teramo, Italy; 7 Eli Lilly and Company, Indianapolis, IN USA; 8 Duke Molecular Physiology Institute, Duke University Durham, Durham, NC USA; 9 Department of Pharmacology and Systems Physiology, University of Cincinnati College of Medicine, Cincinnati, OH USA; 10 Department of Clinical Medicine, Faculty of Health and Medical Sciences, University of Copenhagen, Copenhagen, Denmark; 11 Steno Diabetes Center Copenhagen, Herlev, Denmark; 12 Department of Clinical Epidemiology, Aarhus University, Aarhus, Denmark; 13 Department of Epidemiology, Boston University, Boston, MA USA; 14 Department of Clinical Sciences, Lund University Diabetes Center, Lund University, Malmö, Sweden; 15 Steno Diabetes Center Odense, Odense University Hospital, Odense, Denmark; 16 Department of Clinical Research, University of Southern Denmark, Odense, Denmark; 17 The Children’s Obesity Clinic, accredited European Centre for Obesity Management, Department of Pediatrics, Holbæk Hospital, Holbæk, Denmark; 18 Faculty of Health and Medical Sciences, University of Copenhagen, Copenhagen, Denmark; 19 Department of Obstetrics and Gynecology, Copenhagen University Hospital Herlev, Herlev, Denmark; 20 Center for Pregnant Women with Diabetes, Rigshospitalet, Copenhagen, Denmark; 21 Department of Obstetrics, Rigshospitalet, Copenhagen, Denmark; 22 Center for Clinical Metabolic Research, Department of Medicine, Gentofte Hospital, Copenhagen, Denmark; 23 Center for Clinical Research and Prevention, Copenhagen University Hospital – Bispebjerg and Frederiksberg, Copenhagen, Denmark; 24 Department of Physiology & Pharmacology, Karolinska Institutet, Stockholm, Sweden; 25 Dr Margarete Fischer-Bosch Institute of Clinical Pharmacology, Stuttgart, Germany; 26 University of Tübingen, Tübingen, Germany

**Keywords:** Functional genomics, Obesity, Metabolism, Endocrine system and metabolic diseases

## Abstract

Incretin-based therapies are highly successful in combatting obesity and type 2 diabetes^1^. Yet both activation and inhibition of the glucose-dependent insulinotropic polypeptide (GIP) receptor (GIPR) in combination with glucagon-like peptide-1 (GLP-1) receptor (GLP-1R) activation have resulted in similar clinical outcomes, as demonstrated by the GIPR–GLP-1R co-agonist tirzepatide^2^ and AMG-133 (ref. ^3^) combining GIPR antagonism with GLP-1R agonism. This underlines the importance of a better understanding of the GIP system. Here we show the necessity of β-arrestin recruitment for GIPR function, by combining in vitro pharmacological characterization of 47 *GIPR* variants with burden testing of clinical phenotypes and in vivo studies. Burden testing of variants with distinct ligand-binding capacity, Gs activation (cyclic adenosine monophosphate production) and β-arrestin 2 recruitment and internalization shows that unlike variants solely impaired in Gs signalling, variants impaired in both Gs and β-arrestin 2 recruitment contribute to lower adiposity-related traits. Endosomal Gs-mediated signalling of the variants shows a β-arrestin dependency and genetic ablation of β-arrestin 2 impairs cyclic adenosine monophosphate production and decreases GIP efficacy on glucose control in male mice. This study highlights a crucial impact of β-arrestins in regulating GIPR signalling and overall preservation of biological activity that may facilitate new developments in therapeutic targeting of the GIPR system.

## Abstract (web summary)

Molecular pharmacological characterization and association testing of human *GIPR* genetic variants with follow-up analysis in mice shows that β-arrestins regulate GIPR signalling and thereby strongly contribute to metabolic outcomes.

## Main

Several naturally occurring G protein-coupled receptor (GPCR) genetic variants have been shown to affect receptor function and to result in undesired phenotypic traits and unintended responses to clinically used pharmaceuticals, a third of which target GPCRs^4,5^. Studying receptor genetic variability can provide a deeper understanding of the physiological roles of a given system. Optimally, such studies cover allelic series comprising both loss-of-function (LoF) variants and gain-of-function (GoF) variants, thereby strengthening the mechanistic interpretation of changed gene function. Genetic variants of glucose-dependent insulinotropic polypeptide receptor (GIPR) gene, including the common variant (E354Q) and two rare LoF variants (R190Q and E288G), have been associated with changes in cardiometabolic traits^6–11^. Yet there is a lack of comprehensive characterization of genetic variations in the *GIPR* to understand the role of GIPR signalling in human (patho)physiology.

In this Letter, we sequenced *GIPR* in four Danish study cohorts (*N* = 10,523), including patients newly diagnosed with type 2 diabetes (T2D) (DD2 cohort), individuals from a population-based study cohort (Inter99 cohort) screened for T2D, a cohort of obese children and adolescents (Holbaek Study) and a cohort of women with gestational diabetes mellitus (GDM cohort) in search for genetic variants in the GIPR. Of the identified 61 *GIPR* variants, we selected all 47 non-synonymous variants for further functional characterization (Supplementary Table 1).

**Fig. 1:**
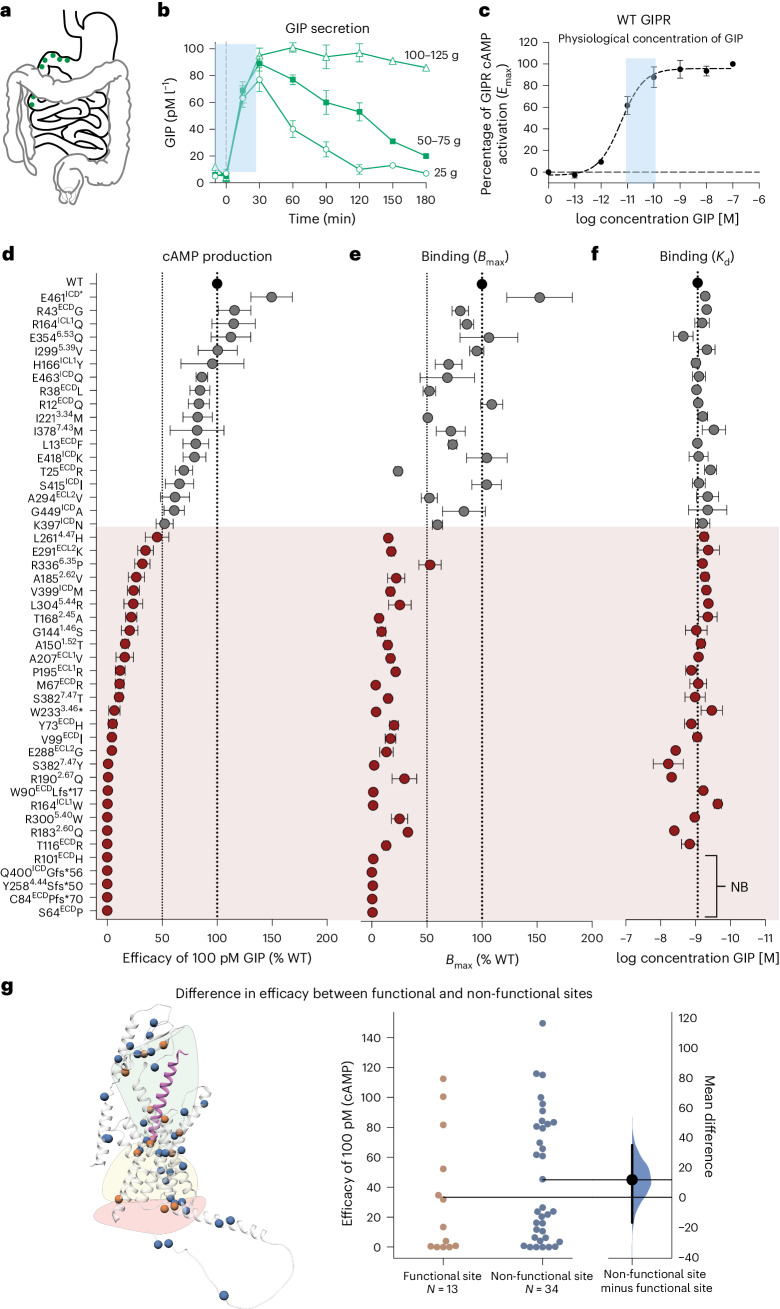
cAMP production and binding properties of the 47 non-synonymous *GIPR* variants. **a**, Localization of GIP-secreting cells in the gastrointestinal tract. **b**, GIP secretion in response to ingestion of low (25 g), moderate (50–75 g) or high (100–125 g) amounts of glucose according to previous human studies (*N* = 8)^12,13^. The blue box highlights the range from low (10 pM) to high (100 pM) levels of GIP. **c**, The corresponding area of the physiological range of GIP on a WT GIPR dose-response curve for cAMP production. **d**, cAMP production at 100 pM GIP, shown as percentage of activity compared with WT GIPR. Grey, WT-like activity and dark red, below 50% activity of WT. The data represent the mean ± s.e.m. WT GIPR: *N* = 18; R12Q, S64P, Y73H, R164Q, P195R–A207V, S382T–S382Y, S415I–G449A: *N* = 6; L13F–R38L, V99I–R101H, R164W, T168A, L261H, E461*–E463Q: *N* = 4; R43G, M67R, C84P–W90L, I221M, R336P: *N* = 5; T116R–A150T, H166Y, R183Q–A185V, W233*–Y258S, E291K–R300W, E354Q–I378M, K397N–V399M: *N* = 3; R190Q: *N* = 9; E288G: *N* = 8; L304R: *N* = 7. Independent experiments were performed in duplicate. **e**,**f**, Homologous competition binding of GIP, showing maximum binding capacity (*B*_max_) and affinity (*K*_d_) compared with WT GIPR. The variants are arranged in the same order as in **b**. NB, no binding. The data represent the mean ± s.e.m. WT GIPR: *N* = 18; R12Q: *N* = 5; L13F–R101H, G144S–A207V, W233*, E288G, E354Q, S382T–G449A, E463Q: *N* = 3; T116R, I221M, Y258S–L261H, E291K–R336P, I378M, E461*: *N* = 4. Independent experiments were performed in duplicate. **g**, Illustration of the GIPR with variants located at functional sites (orange) and non-functional sites (blue). Green area: ligand binding site; yellow area: (micro) switches; red area: G protein interface (left). Mean difference in cAMP efficacy between variants at functional (*N* = 13) and non-functional (*N* = 34) sites (right). No significant difference was observed between the efficacy of 100 pM, *P* = 0.30. Statistical significance was assessed using the Wilcoxon rank-sum test (two sided). Source data

**Extended Data Fig. 1:**
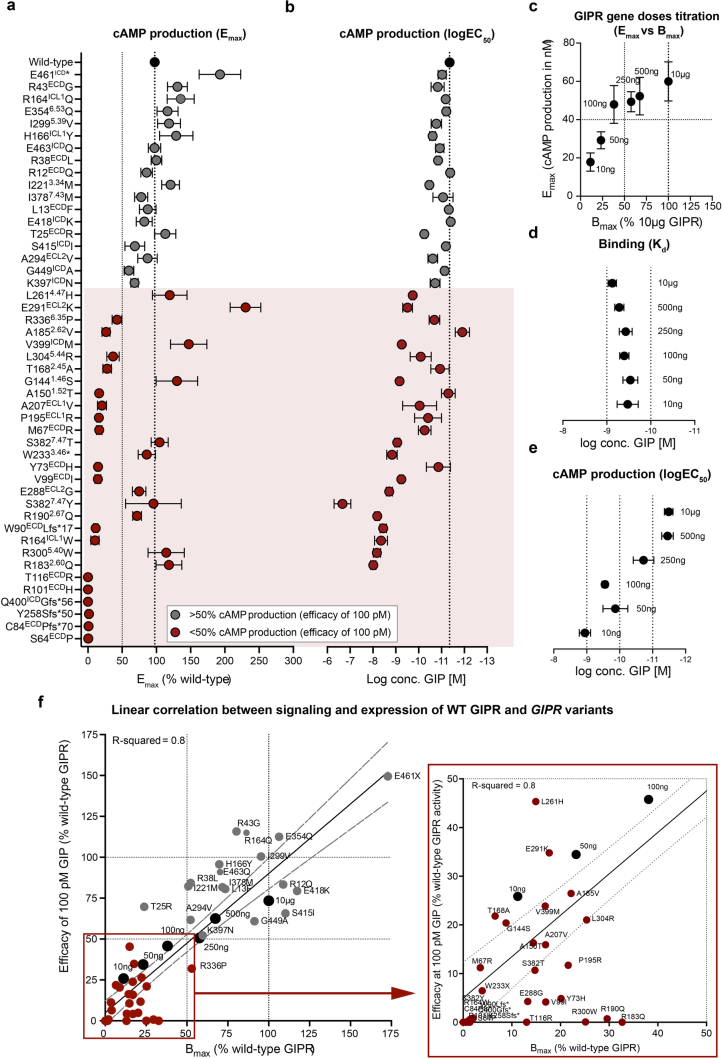
Maximal signalling and potency of the 47 *GIPR* variants in cAMP production, and the relation between WT GIPR gene expression and signalling. **a**, cAMP production *E*_max_, shown as percentage of WT GIPR activity. The variants are arranged according to the cAMP production (efficacy at 100 pM GIP). Grey, WT-like; dark red, below 50% activity. **b**, cAMP production potency (logEC_50_). **c**, *E*_max_ (cAMP production) vs *B*_max_ (homologues competitive binding) of WT GIPR, expressed at DNA doses ranging from 10 ng to 10 µg. **d**, GIP binding affinity (*K*_d_) of WT GIPR at DNA doses ranging from 10 ng to 10 µg. **e**, Potency (logEC_50_) of WT GIPR at DNA doses ranging from 10 ng to 10 µg in cAMP production. **f**, Linear correlation between WT GIPR expression and signalling of the 47 *GIPR* variants. Expression was measured as *B*_max_, and signalling was measured as cAMP production efficacy at 100 pM. The data represent the mean ± s.e.m. of a minimum of *N* = 3. Independent experiments were performed in duplicate. Source data

First, we measured GIP-mediated Gs activity via cyclic adenosine monophosphate (cAMP) accumulation against the reference human wild-type (WT) GIPR and determined their maximal signal (*E*_max_) and GIP potency (EC_50_) (Extended Data Fig. 1a,b). We used a range of GIP concentrations corresponding to those observed in response to ingestion of low, moderate and high amounts of glucose as measured in earlier clinical studies^12,13^ (Fig. 1a,b). Thus, ‘low’ corresponded to ~10 pM and ‘high’ to ~100 pM (Fig. 1c). Next, we ranked the 47 variants by their signalling activity at 100 pM GIP and identified 29 variants with an activity <50% of WT. These were classified as partial and complete LoF variants (referred to as LoF variants hereafter) (Fig. 1d), with a cumulative allele frequency of 1% in the combined Danish cohorts. The binding capacity of GIP (*B*_max_) was impaired for all 29 LoF variants (Fig. 1e). Five showed undetectable GIP binding (S64P, C84Pfs*70, R101H, Y258Sfs*50 and Q400Gfs*56; Fig. 1f). For the remaining 24 variants, high affinity (*K*_d_) for GIP was retained (Fig. 1f), suggesting a loss of signal due to low receptor expression. Four variants (T116R, R183Q, R190Q and R300W) with maintained *B*_max_ displayed no cAMP signalling at 100 pM GIP, unlike variants with comparable *B*_max_, for example, G144S, A185V and L304R (Fig. 1d,e). At higher GIP doses, cAMP production was restored in R183Q, R190Q and R300W, while T116R remained unable to signal up to 100 nM GIP (Extended Data Fig. 1a), suggesting these residues are critical for G protein interaction. Conversely, the T25R variant maintained over 50% signalling capacity at a 25% *B*_max_, indicating a possible structural modification that enhances signalling despite lower GIP binding. Gene dose titration experiments were performed to investigate the impact of low receptor expression on GIPR signalling. WT GIPR ranging from doses of 10 ng to 10 µg (1,000-fold increase) receptor DNA revealed an association between GIPR signalling and cell surface expression (Extended Data Fig. 1c). As expected, equal binding affinity of GIP was observed independently of receptor expression levels. In contrast, a right shift towards lower potency was observed with decreasing GIPR levels (Extended Data Fig. 1d,e). Combining the data for receptor expression and signalling at 100 pM GIP of the 47 *GIPR* variants as well as the WT GIPR transfected with increasing DNA doses, a linear correlation (*r*^2^ = 0.8) appeared (Extended Data Fig. 1f). This suggests that receptor surface expression is the main driver of activity of the *GIPR* variants, similar to what has been observed in the glucagon-like peptide-1 receptor (GLP-1R)^14^.

**Extended Data Fig. 2:**
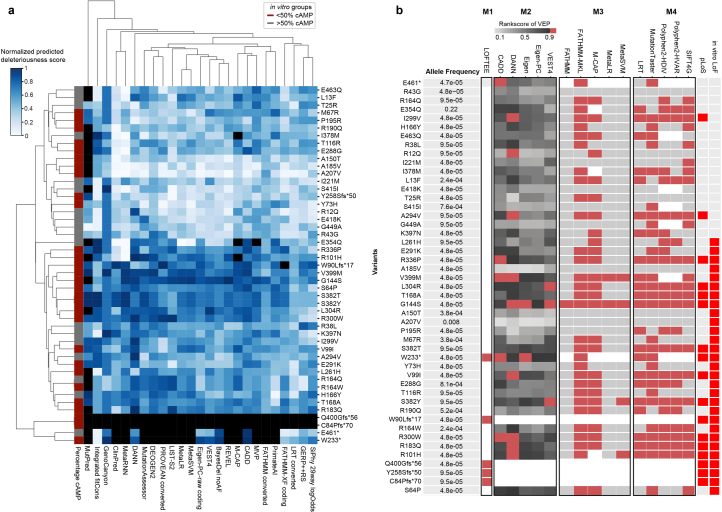
Variant effect predictor (VEP) correlations to laboratory-tested *GIPR* variants. **a**, Cluster heatmap of the VEP normalized predicted deleteriousness scores, ranging from benign (0) to deleterious (1). The x-axis shows the included VEPs, and the classification of the laboratory-tested variants according to cyclic adenosine monophosphate (cAMP) accumulation (*left*). **b**, Prediction of variants potentially leading to a loss-of-signalling (LoS) molecular phenotype with four VEP masks (M1–M4)^15^. The 47 *GIPR* variants and their allele frequencies in the Danish population (all four study cohorts combined) are shown in the left panel, sorted according to their cAMP production (from low (bottom) to high (top)) compared with that of WT (Fig. 1d). M1, the Loss-of-Function Transcript Effect Estimator (LOFTEE) high-confidence (HC) mask with predicted LoS variants (red). M2–M4, grouped VEPs. A variant was considered a LoS variant if all VEPs in one of the four masks predicted the variant as a LoS variant (red). ‘*pLoS*’ represents the variants predicted to have a LoS molecular phenotype (bright red). ‘*in vitro* LoF’ represents the tested *GIPR* LoF variants with < 50% cAMP production (bright red). *NA*s are white. In total, 17 variants were pLoS variants, of which 15 overlapped with the *in vitro* cAMP LoF variants, and two were *in vitro* cAMP WT-like variants. Source data

The 29 LoF mutations were evenly distributed across the GIPR structure (Fig. 1g), which led us to speculate whether variants located at functional sites were more deleterious than those at non-functional sites. Thus, we mapped the location of variants to the ligand binding interface, the G protein interface and the activity-regulating (micro-)switches, which revealed a comparable cAMP production between variants located at functional sites (*N* = 13) and those at non-functional sites (Wilcoxon rank-sum test *P* = 0.3) (Fig. 1g). Variant effect predictors (VEPs) did not reveal any correlations between measured efficacy and VEP scores (mean *r*^2^ across 15 predictors: 0.09) (Extended Data Fig. 2a). For previously described VEP masks^15^, we observed an under-representation of variants predicted to cause impaired signalling compared with the in vitro LoF variants (Extended Data Fig. 2b). Hence, variant location and VEPs are insufficient for estimating nuanced *GIPR* variant outcomes in vitro.

**Fig. 2:**
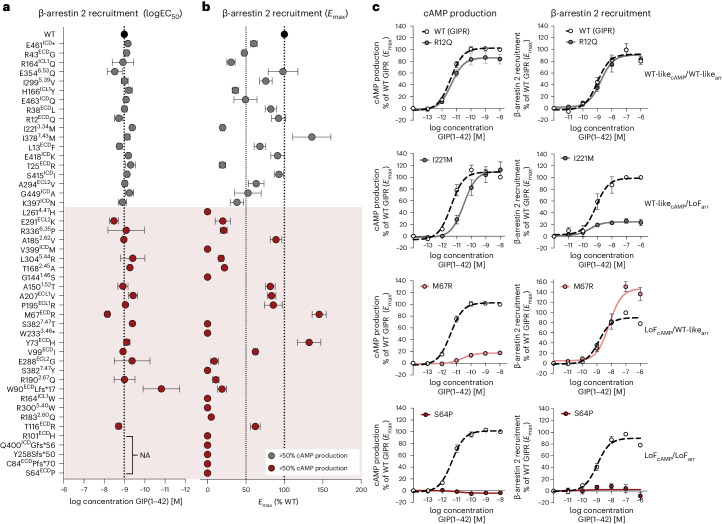
β-arrestin 2 recruitment profiles of *GIPR* non-synonymous variants. **a**, β-arrestin 2 recruitment potency (logEC_50_) of GIP. Variants are arranged according to cAMP efficacy of 100 pM GIP. **b**, β-arrestin 2 recruitment maximal signalling (*E*_max_), shown as a percentage of WT GIPR activity. NA, no activation. **c**, Dose-response curves of cAMP production (left) and β-arrestin 2 recruitment (right) of *GIPR* variants R12Q, I221M, M67R and S64P, respectively. Data represents the mean ± s.e.m. WT GIPR: N = 30; R12Q–S64P, Y73H–C84P, V99I–T168A, A185V, P195R: *N* = 5; M67R: *N* = 6; W90L, R183Q, A207V, R336P–E354Q: *N* = 4; R190Q, E288G: *N* = 6; I221M–L261H, E291K–L304R, I378M–E463Q: *N* = 3. Independent experiments were performed in duplicate. Source data

Given that GIP binding leads to receptor phosphorylation by GPCR kinases, resulting in sustained recruitment of β-arrestins^16^, we determined β-arrestin recruitment for the 47 non-synonymous *GIPR* variants. Since the WT GIPR predominantly recruits β-arrestin 2 over β-arrestin 1 (ref. ^17^), we focused on β-arrestin 2 in this study (henceforth, β-arrestin 2 will be referred to as β-arrestin). We observed no major differences in potency for most variants, apart from a few lacking the ability to recruit β-arrestin (Fig. 2a). The variants with maintained β-arrestin recruitment displayed varying *E*_max_ (Fig. 2b), falling into four main groups. Of the 29 variants with impaired cAMP production, 21 also had impaired β-arrestin recruitment, hereafter classified as LoF_cAMP_/LoF_arr_ (defined as <50% activity in either pathway). Eight variants (M67R, Y73H, V99I, T116R, A150T, A185V, P195R and A207V) with cAMP production LoF showed WT-like β-arrestin recruitment (LoF_cAMP_/WT-like_arr_). Six variants (T25R, R43G, R164Q, H166Y, I221M and K397N) showed maintained cAMP production but low β-arrestin recruitment (WT-like_cAMP_/LoF_arr_). A fourth group of ten variants (R12Q, L13F, R38L, A294V, I299V, E354Q, S415I, E418K, G449A and E463Q) were WT-like (WT-like_cAMP_/WT-like_arr_). The dose-response relationship in cAMP production versus β-arrestin recruitment of four variants (R12Q, S64P, M67R and I221M), typical of each of the four characterized groups, illustrated the distinct signalling profiles of the variants (Fig. 2c). Beyond these four groups, one variant (I378M) showed more β-arrestin recruitment (GoF) with WT-like cAMP production (WT-like_cAMP_/GoF_arr_) and another (E461*) had more cAMP production (GoF) and preserved β-arrestin recruitment (GoF_cAMP_/WT-like_arr_). Structural mapping revealed that variants with similar signalling preferences did not cluster in specific regions or domains (Supplementary Fig. 1), except for the LoF_cAMP_/WT-like_arr_. In this group, all variants except A185V were located in the extracellular domain (M67R, Y73H, V99I and T116R) and extracellular loop 1 (P195R and A207V), indicating that mutations in these areas specifically affected cAMP production.

**Extended Data Fig. 3:**
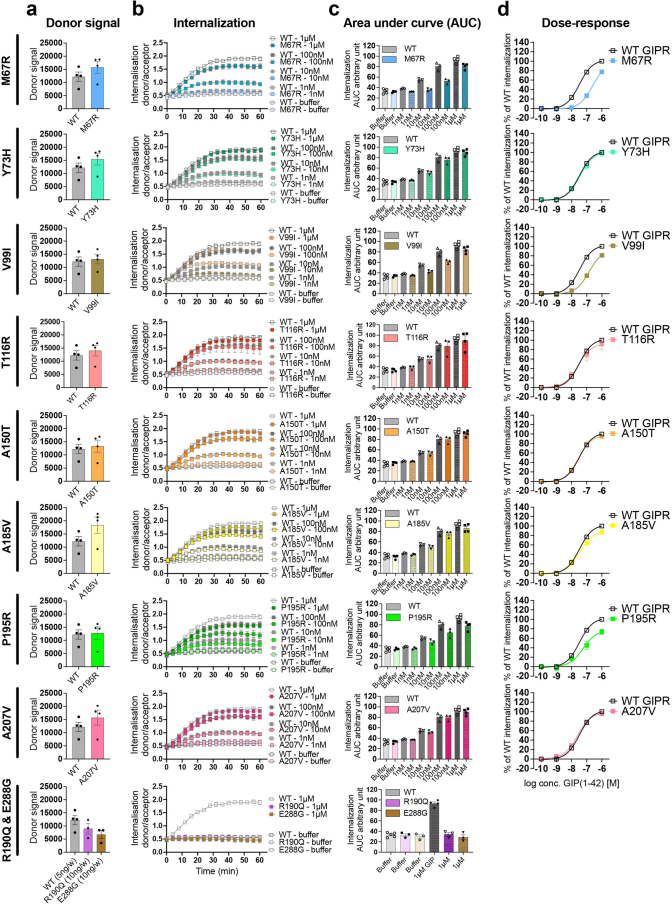
Internalization abilities of the eight LoF_cAMP_/WT-like_arr_ variants and two LoF_cAMP_/LoF_arr_. **a**, The donor signal represents an estimate of receptor surface expression of the cells transfected with SNAP-WT GIPR or variants. *N* = 4. **b**, Internalization ratios of the SNAP-GIPR variants over time following stimulation with GIP in doses ranging from 1 nM to 1 µM. *N* = 4. **c**, Area under the curves of the SNAP-WT GIPR vs. SNAP-GIPR variants in response to GIP stimulation in doses ranging from 1 nM to 1 µM. *N* = 4. **d**, Dose-response curves of maximal internalization of the SNAP-GIPR variants, shown as percentage of maximal SNAP-WT GIPR internalization. *N* = *4*. Data are shown as the mean ± s.e.m. Independent experiments were performed in triplicates. Source data

Given the necessity of β-arrestin recruitment for GIPR internalization^16^, we probed the internalization profile of the eight LoF_cAMP_/WT-like_arr_ variants together with the two LoF_cAMP_/LoF_arr_ variants, R190Q and E288G (previously published to be impaired in internalization^11^). All LoF_cAMP_/WT-like_arr_ variants demonstrated preserved internalization capabilities (Extended Data Fig. 3a,b). Of these, Y73H, T116R, A150T, A185V and A207V had nearly identical levels of internalization as the WT GIPR, while M67R, V99I and P195R displayed a slightly diminished internalization (Extended Data Fig. 3c,d). Conversely, the two LoF_cAMP_/LoF_arr_ variants, R190Q and E288G, with maintained GIP binding but impaired cAMP signalling and β-arrestin recruitment, did not internalize, highlighting the importance of β-arrestin recruitment but not Gs activation for GIPR internalization.

**Fig. 3:**
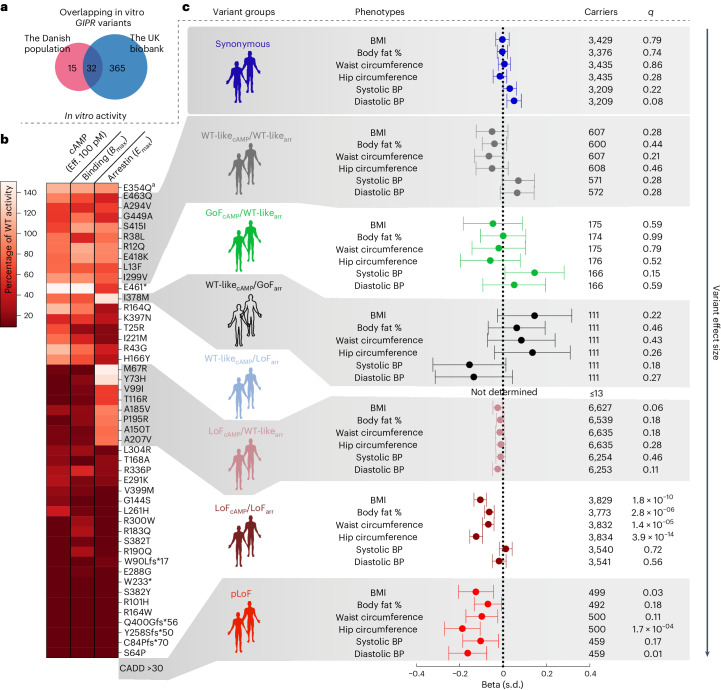
Binding- and signalling-based grouping of *GIPR* variants and their respective burden association on cardiometabolic phenotypes in the UK Biobank. **a**, The overlap between non-synonymous *GIPR* variants found in the Danish population and the UK Biobank. *N*, total cohort sample size. **b**, The pharmacological profile for all *GIPR* variants in terms of cAMP accumulation (efficacy (Eff.) at 100 pM GIP), binding ability and β-arrestin recruitment compared with WT receptor clustered into six in vitro phenotype groups. **c**, A forest plot showing burden test statistics in the UK Biobank cohort (*N* = ~440,000). Carriers represent the number of individuals for each phenotype carrying any of the *GIPR* variants within that *GIPR* variant group. *q*, FDR-adjusted *P* values of the burden tests (performed using ACAT-O). pLoF denotes variants predicted to cause loss of function with VEP^47^ and/or CADD score > 30, that is, pLoF variants were not functionally characterized. ^a^The WT-like_cAMP_/WT-like_arr_ variant, E354Q, was excluded from burden testing due to high MAF (~20%). Beta represents the effect size as standard deviation of the phenotype with error bars representing the 95% CI. Source data

**Extended Data Fig. 4:**
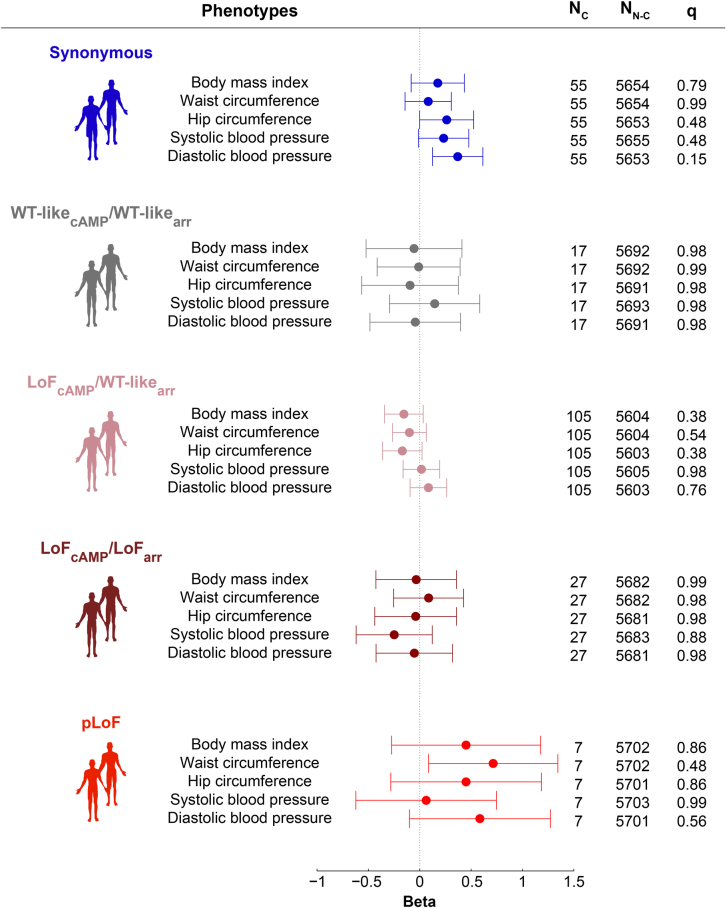
Burden association of the *GIPR* variant groups on cardiometabolic phenotypes in the Danish cohorts. Forest plot showing burden test statistics in the Danish population (Inter99). *N*_C_, number of individuals for each phenotype carrying any of the variants within that variant group. *N*_N-C_, number of individuals not carrying any of the variants within that variant group. q, false discovery rate (FDR) adjusted *p*-values of the burden tests (performed using SKAT/SKAT-O). pLoF denotes variants predicted to cause loss of function with VEP. pLoF variants were not functionally characterized but include some of the LoF_cAMP_/LoF_arr_ variants. The E354Q variant was excluded from burden testing of the WT-like_cAMP_/WT-like_arr_ group due to high minor allele frequency (MAF; ~20%). Beta and error bars represent effect size as standard deviation and the 95% confidence interval, respectively, derived from a linear model. Source data

**Extended Data Fig. 5:**
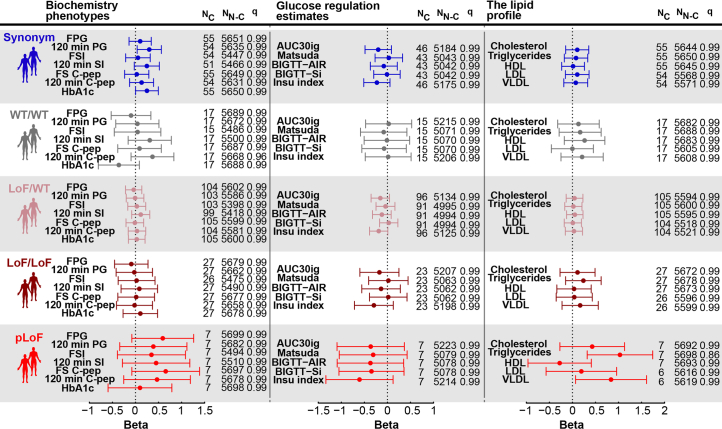
Forest plots of burden tests of cardiometabolic phenotypes in the Danish population (Inter99). Association results of metabolic-associated biochemistry measures, estimates of beta cell function and insulin sensitivity, and cardiovascular-associated phenotypes (the lipid profile) for all variant groups are shown. Synonym, WT/WT, LoF/WT, LoF/LoF, and pLoF represent the groups of synonymous, WT-like_cAMP_/WT-like_arr_, LoF_cAMP_/WT-like_arr_, LoF_cAMP_/LoF_arr_, and predicted LoF variants, respectively. The E354Q variant was excluded from burden testing of the WT-like_cAMP_/WT-like_arr_ group due to high minor allele frequency (MAF; ~20%). Abbreviations: FPG, fasting plasma glucose levels; PG, plasma glucose levels; FSI, fasting serum insulin levels; SI, serum insulin levels; FS C-pep, fasting serum C-peptide levels; HbA1c, hemoglobin A1c; AUC30ig, area under curve 0–30 min insulin/glucose; BIGTT-AIR, beta cell function insulin sensitivity glucose tolerance test (BIGTT)-acute insulin response (insulin secretion); BIGTT-SI, BIGTT-sensitivity index (insulin sensitivity); Insu index, insulinogenic index (insulin secretion); HDL, high-density lipoprotein; LDL, low-density lipoprotein; VLDL, very low-density lipoprotein. *N*_C_ represents the number of individuals for each phenotype carrying any of the variants within that variant group. *N*_N-C_ represents the number of individuals not carrying any of the variants within that variant group. *q*, FDR adjusted *p*-values of the burden tests (performed using SKAT/SKAT-O). Beta and error bars represent effect size as standard deviation and the 95% confidence interval, respectively, derived from a linear model. Source data

**Extended Data Fig. 6:**
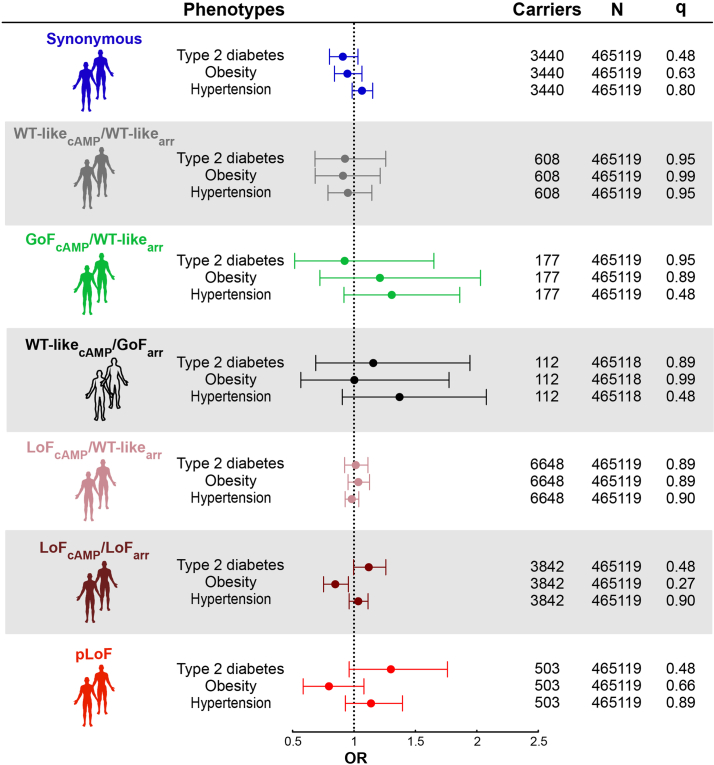
Forest plot of binary phenotype burden tests in the UK Biobank population. For each GIPR variant group, the odds ratios (OR) are shown for binary traits with corresponding confident intervals. ‘*Carriers*’ represent the number of individuals for each phenotype carrying any of the *GIPR* variants within that GIPR variant group. We excluded the E354Q variant from the burden testing of the WT-like_cAMP_/WT-like_arr_ group due to high minor allele frequency (MAF; ~20%). ‘*N*’ represents the total number of individuals for each phenotype. *q*, the false discovery rate (FDR) adjusted *p*-values of the burden tests (performed using ACAT-O). Error bars represent the 95% confidence interval of the OR. Source data

**Extended Data Fig. 7:**
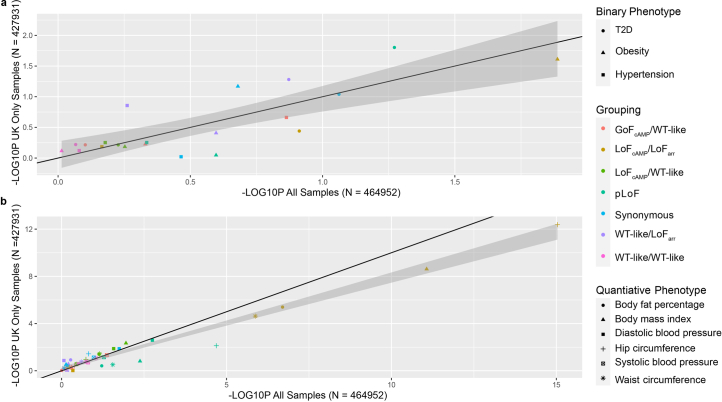
Comparison of *p*-values between with all UK Biobank samples or only individuals from the United Kingdom. -log_10_(*P*) for each phenotype/grouping combination when testing either all samples passing quality control or limited to those with United Kingdom (UK) ancestry. The x-axis shows *p*-values (derived from burden testing using ACAT-O) for testing of all 465,000 individuals (Source Data of Fig. 3 and Extended Data Fig. 6). The y-axis shows *p*-values (derived from burden testing using ACAT-O) for testing of only 428,000 UK individuals. **a**, Binary traits. Circles represent type 2 diabetes (T2D), triangles represent obesity, and squares represent hypertension. Error bands represent the 95% confidence interval (CI) of the regression line. **b**, Quantitative traits. Circles, triangles, squares, pluses, crossed squares, and asterisks represent body fat percentage, body mass index, diastolic blood pressure, hip circumference, systolic blood pressure, and waist circumference, respectively. Error bands represent 95% CI of the regression line. Source data

**Extended Data Fig. 8:**
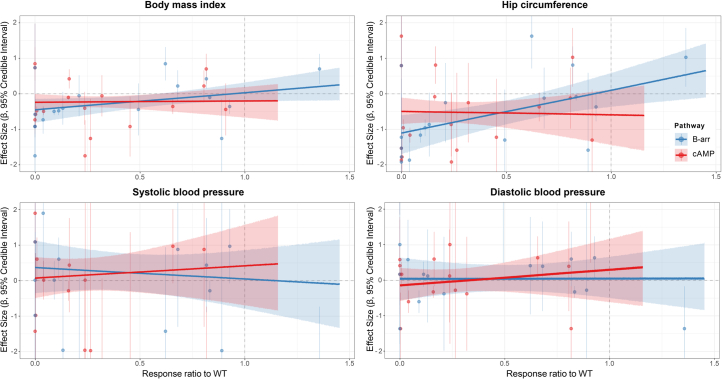
Meta-analysis regression of single-variant phenotypic association estimates on cAMP signalling and β-arrestin recruitment. UK Biobank single-variant phenotypic association effect sizes (see Fig. 3) regressed on each variant’s *in vitro* effect on cAMP production (see Fig. 1) and β-arrestin recruitment (see Fig. 2) compared to WT GIPR. Analysis performed as a linear interaction model of phenotypic effect size estimates (y-axis) as response of single-variant (points) β-arrestin recruitment (B-arr, blue) and cAMP signalling (red) impact (x-axis). We used Bayesian Gaussian model which accounts for uncertainty in both individual phenotype estimates (vertical bars as standard error of the beta (*β*) from the linear regression model) and *in vitro* uncertainty (not shown). Error bands represent 95% credible interval and grey dashed lines represent no effect. Source data

Next, we sought to link the molecular pharmacology of the *GIPR* variants to cardiometabolic phenotypes using Danish cohorts (T2D: DD2 and Inter99, *N* = 7,173 and quantitative traits: Inter99, *N* = 5,711) and the UK Biobank^18^ (465,506 whole-exome-sequenced participants). We detected 365 non-synonymous *GIPR* variants in the UK Biobank (Supplementary Table 2), of which 32 of the 47 Danish non-synonymous *GIPR* variants were represented (Fig. 3a). We grouped the characterized variants according to their molecular characteristics (Fig. 3b) to perform burden testing. Additionally, we performed burden testing of all synonymous and predicted LoF (pLoF) *GIPR* sequenced variants in the Danish population and the UK Biobank, respectively (Supplementary Tables 1 and 2), which were not functionally characterized. We did not detect significant associations with any phenotype for any of the *GIPR* variant groups in the Danish cohorts (Extended Data Figs. 4 and 5, Supplementary Table 3 and Supplementary Fig. 2), probably due to low power given the few carriers in all variant groups. In the UK Biobank, ~3,800 carriers of LoF_cAMP_/LoF_arr_ variants displayed lower body mass index (BMI) (effect size (*β*) = −0.10 s.d., 95% confidence interval (CI) −0.13 to −0.08), body fat percentage (*β* = −0.06 s.d., 95% CI −0.09 to −0.04), hip circumference (*β* = −0.12 s.d., 95% CI −0.15 to −0.09) and waist circumference (*β* = −0.07 s.d., 95% CI −0.09 to −0.04) than non-carriers (Fig. 3c). The LoF_cAMP_/LoF_arr_ carriers also had a nominally lower odds ratio (OR) for obesity compared to non-carriers (OR 0.85, CI 0.75 to 0.90; Extended Data Fig. 6), agreeing with the adiposity protective effect of impaired GIPR signalling^9–11^. Similarly, carriers of the 105 pLoF variants found in the UK Biobank (488 carriers) displayed significantly lower hip circumference (*β* = −0.19 s.d., 95% CI −0.27 to −0.11) and slightly lower BMI (*β* = −0.13 s.d., 95% CI −0.21 to −0.04; Fig. 3c). In contrast to the LoF_cAMP_/LoF_arr_ variants, carriers of the pLoF variants also showed slightly lower diastolic blood pressure (*β* = −0.16 s.d., 95% CI −0.25 to −0.08; Fig. 3c) with no increase in hypertension (Extended Data Fig. 6). Intriguingly, the lower adiposity phenotypes in LoF_cAMP_/LoF_arr_ carriers were not found in the ~6,600 LoF_cAMP_/WT-like_arr_ carriers (Fig. 3c). Likewise, no associations were observed in the other *GIPR* variant groups (Fig. 3c and Extended Data Fig. 6). Comparable results were found when restricting to 427,931 individuals of British ancestry (Extended Data Fig. 7). Finally, we conducted a meta-analysis regression using single-variant estimates of *GIPR* variants regressed on their molecular phenotype, revealing an impact of impaired β-arrestin recruitment (below 50% of WT) on BMI and hip circumference but not on blood pressure and no impact of impaired cAMP production (Extended Data Fig. 8).

**Fig. 4:**
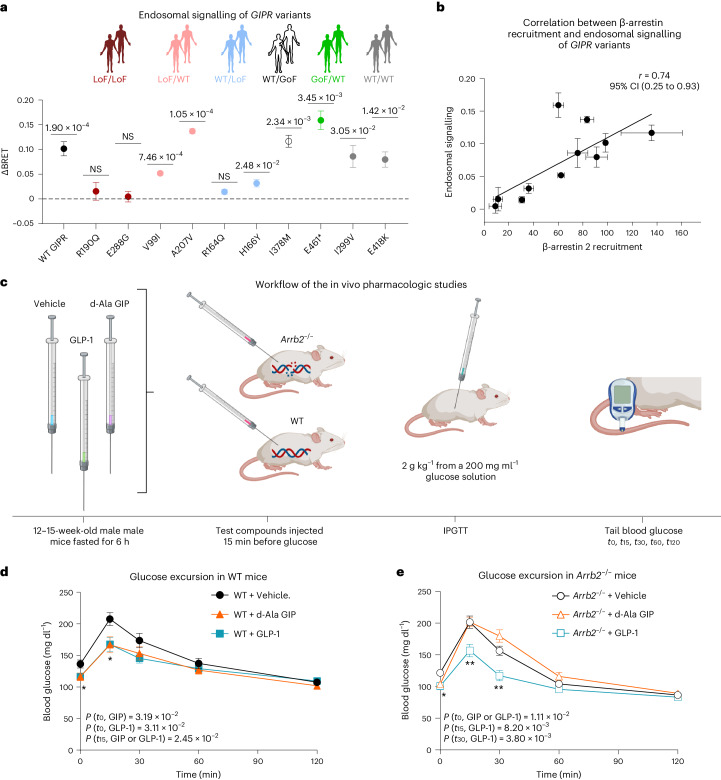
Impact of β-arrestin on endosomal signalling of *GIPR* variants and glucose excursion in male mice. **a**, Gs pathway engagement at early endosomes of representative *GIPR* variants from each of the six in vitro-based groups. The delta (Δ) BRET values are calculated by subtracting the vehicle value from the top values (1 µM) and evaluated by a one sample *t*-test (two-sided) performed on the mean of individual experiments with hypothetical value set to 0. The data represent mean ± s.e.m. of WT GIPR, *N* = 8 and *GIPR* variants, *N* = 4. Independent experiments were performed in duplicates. Exact *P* values are shown above the data points for variants significantly different from 0. **b**, Pearson correlation analysis of endosomal signalling (ΔBRET) with β-arrestin 2 recruitment (*E*_max_ % of WT) of the variants. LoF/LoF (LoF_cAMP_/LoF_arr_), LoF/WT (LoF_cAMP_/WT-like_arr_), WT/LoF (WT-like_cAMP_/LoF_arr_), WT/GoF, (WT-like_cAMP_/GoF_arr_), GoF/WT (GoF_cAMP_/WT-like_arr_) and WT/WT (WT-like_cAMP_/WT-like_arr_). The data represent the mean ± s.e.m. Independent experiments were performed in duplicates. The number of replicates is the same as Figs. 4a and 2. **c**, Workflow of the pharmacologic studies in *Arrb2* knockout (KO) mice. *t*_0_, *t*_15_, *t*_30_, *t*_60_ and *t*_120_ represent timepoints (in minutes) after initiating an intraperitoneal glucose tolerance test (IPGTT). **d**,**e**, Glucose excursion during an IPGTT in WT male mice (*N* = 10–14 per group) (**d**) and male *Arrb2* KO mice (*N* = 12–13 per group) (**e**) 15 min following vehicle (black circles), d-Ala GIP (orange triangles) or GLP-1 (teal squares). Mouse data are represented as mean ± s.e.m. Statistical significance was determined by two-way analysis of variance, and Holm-Šidák testing was used to correct for multiple testing. The *P* value for either GLP-1 or GIP compared with vehicle is shown. Figure 4c was created with BioRender.com. Source data

These findings illustrate that differential GIPR signalling/coupling may contribute to altered phenotypic outcomes and underscore the importance of β-arrestin and GIPR internalization for proper function. This prompted us to further assess the role of β-arrestin for GIPR signalling by investigating the endosomal signalling in representative *GIPR* variants from each of the six in vitro*-*based groups. We coupled mini Gs (mGs, an engineered G protein that detects the active receptor component for engaging and activating heterotrimeric Gs) with enhanced bystander bioluminescence resonance energy transfer (BRET) at early endosomes following exposure to GIP^19–21^. Variants with diminished β-arrestin recruitment displayed minimal to no signalling from early endosomes. In contrast, those that retained β-arrestin recruitment effectively recruited mGs to early endosomes and continued their intracellular signalling to varying degrees, compared with the WT GIPR (Fig. 4a). These data demonstrate the crucial role of β-arrestin in sustaining Gs-mediated signalling of the GIPR from endosomes, supported by the positive correlation between β-arrestin recruitment and GIP-induced endosomal signalling (Fig. 4b). Additionally, we investigated the basal subcellular localization of the variants in relation to their distribution at the plasma membrane, endosomes, Golgi apparatus and endoplasmic reticulum (ER). Most variants showed distribution patterns similar to the WT GIPR, predominantly at the plasma membrane (Supplementary Fig. 3a). However, only the two LoF_cAMP_/LoF_arr_ variants, R190Q and E288G, deviated slightly from WT GIPR as they appeared to be trapped intracellularly in the ER with reduced expression on the plasma membrane (Supplementary Fig. 3b), which is in line with their overall impaired receptor function.

**Extended Data Fig. 9:**
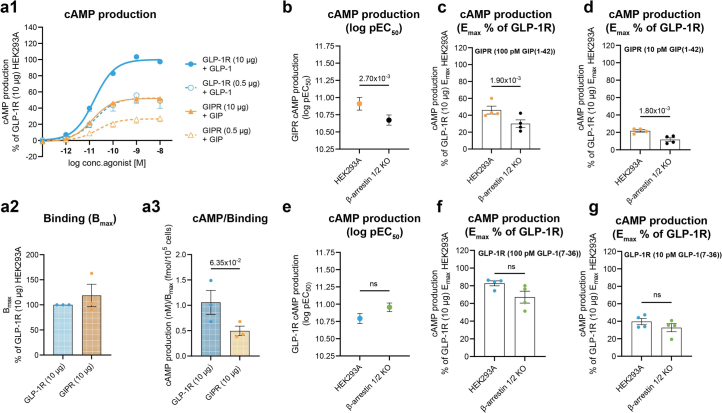
*In vitro* comparison of parental HEK293A cells and β-arrestin 1/2 KO cells transfected with GIPR or GLP-1R. **a1**, cAMP accumulation measured in parental HEK293A cells transfected with 10 µg or 0.5 µg of receptor DNA upon cognate ligand stimulation. GLP-1R stimulated by GLP-1 mediates a more efficacious cAMP response at both receptor concentrations compared to GIPR stimulated by GIP. **a2-a3**, Receptor surface expression (*B*_max_) determined by homologous radio-ligand competition binding shows that GLP-1R **a2**, and GIPR **a3**, are expressed to the same extent on the cell surface. The cAMP formed by GLP-1R is approximately double the magnitude compared to GIPR, considering the number of receptors on the surface. **b**, Potency (pEC_50_) of GIP in cells transfected with GIPR. In the absence of β-arrestin, the potency of GIP is significantly decreased (p < 0.01). **c-d**, Corresponding GIPR cAMP formation upon 100 **c**, and 10 pM **d**, GIP stimulation. The absence of β-arrestin leads to a significant decrease in cAMP formation (*p* < 0.01). **e**, Potency (pEC_50_) of GLP-1 in cells transfected with GLP-1R. A slightly higher, yet nonsignificantly, potency of GLP-1 on the GLP-1R was observed in β-arrestin KO cells compared to parental cells. **f-g**, Corresponding cAMP formation upon 100 pM **f**, and 10 pM **g**, GLP-1 stimulation. A nonsignificant reduction in cAMP formation was observed between WT and β-arrestin KO cells. Statistical significance was assessed using a two-tailed paired *t*-test. The data represent the mean ± s.e.m. of *N* = 3 performed in duplicates. Source data

GIP and GLP-1 are mostly known for enhancing glucose-stimulated insulin secretion from the pancreatic β cells^22,23^. We therefore explored the functional relationship between β-arrestin recruitment and GIP-stimulated glucose regulation by conducting pharmacological interventions in a murine genetic global germline β-arrestin 2 (*Arrb2*) knockout (KO) model. Male mice were injected with native GLP-1 and d-Ala GIP—a highly potent GIPR agonist^24^—before an intraperitoneal glucose tolerance test (IPGTT; Fig. 4c). As expected, both GLP-1 and d-Ala GIP significantly lowered blood glucose levels in WT male mice (Fig. 4d). In contrast, only GLP-1 was able to lower blood glucose in male *Arrb2* KO mice (Fig. 4e). Consistent with this, a significantly lower GIPR-mediated cAMP production was observed in β-arrestin_1/2_ KO HEK293 cells compared with parental HEK293 cells (Extended Data Fig. 9a–d). This was not the case for the GLP-1R signalling, which was unaffected by the absence of arrestins (Extended Data Fig. 9e–g). Thus, in agreement with the physiological effects in humans, β-arrestins were critical for maintained GIPR intracellular signalling and for GIP-mediated lowering of blood glucose levels in mice, indicating a functional role of β-arrestins in sustaining GIP, but not GLP-1, action.

In summary, we show that 29 *GIPR* variants with impaired cAMP production exhibited a proportionally lower agonist binding capacity, indicating that an impaired cAMP-mediated receptor activation was caused primarily by low receptor cell surface expression. A similar observation was found for GLP-1R^14^, emphasizing the importance of cell surface expression in canonical GPCR activation. Among the 29 *GIPR* variants, 21 had impaired β-arrestin recruitment (LoF_cAMP_/LoF_arr_), with the affected residues distributed at various locations indicating allosteric mechanisms, converging towards similar molecular phenotypes.

We and others have previously shown a lowering effect on adiposity-associated phenotypes for the two LoF_cAMP_/LoF_arr_ variants, R190Q and E288G^9–11^. This effect was also recapitulated in the UK Biobank for the pooled group of LoF_cAMP_/LoF_arr_ variants. This adiposity-lowering effect was not found with sustained β-arrestin recruitment (LoF_cAMP_/WT-like_arr_), implying a plausible contribution of β-arrestins to GIPR function in the context of metabolic health. Additionally, enhanced β-arrestin recruitment with maintained cAMP accumulation (WT-like_cAMP_/GoF_arr_) was nominally associated with higher adiposity in this cohort. Our meta-analysis regression supported the impact of β-arrestin recruitment on adiposity. However, since the effect sizes were estimated solely from the variants investigated in vitro, the results may be highly underpowered. In contrast to the β-arrestin contribution in current associations, *GLP-1R* variants leading to impaired β-arrestin recruitment do not contribute to the associations between impaired cAMP signalling variants and higher haemoglobin A1c levels and BMI^14^. We also noticed that the *GIPR* variant groups GoF_cAMP_/WT-like_arr_ and WT-like_cAMP_/GoF_arr_ affected the blood pressure and adiposity measures in the opposite effect directions. We did not observe a changed risk of T2D in LoF_cAMP_/LoF_arr_ variant carriers in the UK Biobank, which is in contrast to the worsened insulin resistance and increased risk of T2D reported for the LoF-mimicking *GIPR* variant E354Q^6,7^. The lack of association with T2D risk in the UK Biobank may, in part, be due to known limitations regarding the register-based diabetes phenotypes in the UK Biobank, even though attempts have been made to establish a more solid T2D definition^25,26^. The lack of phenotype associations in the Danish cohorts may be explained by limited statistical power, and thus we cannot exclude an impact of impaired GIPR signalling on these phenotypes in the Danish population. Furthermore, the nuanced influence of lifestyle factors, which contribute to variation in cardiometabolic phenotypes and the efficacy of pharmacological interventions, may partially shape our findings. Nonetheless, our genetic data support the demonstrated lowering effect of diminished GIPR signalling on adiposity in humans and that the burden of rare protein-coding *GIPR* variants affects body adiposity^9–11^.

Our observation that maintained β-arrestin recruitment may be a strong determinant of the clinical phenotypes (lower adiposity in the LoF_cAMP_/LoF_arr_ group but not in the LoF_cAMP_/WT-like_arr_ group) suggests that a preserved GIPR response may be present despite diminished cAMP signalling if the β-arrestin system is intact. We also supported the pivotal role of β-arrestins in GIPR internalization^16,27,28^ by the in vitro findings, that is, the eight LoF_cAMP_/WT-like_arr_ variants maintained their internalization properties in contrast to the two LoF_cAMP_/LoF_arr_ variants (R190Q and E288G). This is in contrast to the GLP-1R, which can internalize independently of β-arrestins^29^. Additionally, the endosomal signalling experiments showed a clear positive correlation with β-arrestin recruitment, confirming the pivotal role of β-arrestins in regulating the post-activation trafficking^30^ and signalling^31^ of the GIPR and thereby potentially contributing to the unchanged biological actions of GIP in the carriers of LoF_cAMP_/WT-like_arr_. We did, however, not see any major changes in subcellular localization of the variants compared with WT GIPR, except for the two LoF_cAMP_/LoF_arr_ variants, R190Q and E288G, which appeared to be trapped in the ER relative to localization at the plasma membrane, explaining their diminished GIP binding and activity. These findings suggest that the altered function of most of the *GIPR* variants might result from changes in receptor conformation or impaired receptor cell surface expression but not altered subcellular receptor distribution.

In agreement with our in vitro data, GIP treatment in vivo had no glucose-lowering effect in male mice lacking β-arrestins, whereas GLP-1 maintained its insulinotropic properties in the β-arrestin KO mice. Conversely, a recent study showed reduced acute GLP-1-mediated glycaemic control in β cell-specific β-arrestin 2 KO female mice, with a similar trend for d-Ala GIP treatment in the same KO mice^32^. However, our study, along with others^33^, demonstrated consistent insulinotropic actions of GLP-1 regardless of β-arrestin 2 in mice. In contrast, GIP-potentiated insulin secretion relied on β-arrestin 2, underscoring distinct signalling preferences of these closely related receptors. Of note, our mouse studies were conducted in global germline β-arrestin 2 KO mice, which aligns more closely with inherited human genetics. However, it should be noted that these findings are solely derived from studies in male mice, raising uncertainty about the translatability to female mice.

Recent studies have comprehensively profiled coding *GLP1R* variants. One study demonstrated no association between variants impairing cAMP signalling and cardiometabolic phenotypes^34^. However, upon inclusion of expression, a recent study revealed an association between reduced cell surface expression and metabolic phenotypes^14^. Likewise, *GLP1R* variants have been suggested to affect the outcome of GLP-1R agonist treatment^35^, highlighting the importance of genetic influences on drug responses.

**Extended Data Fig. 10:**
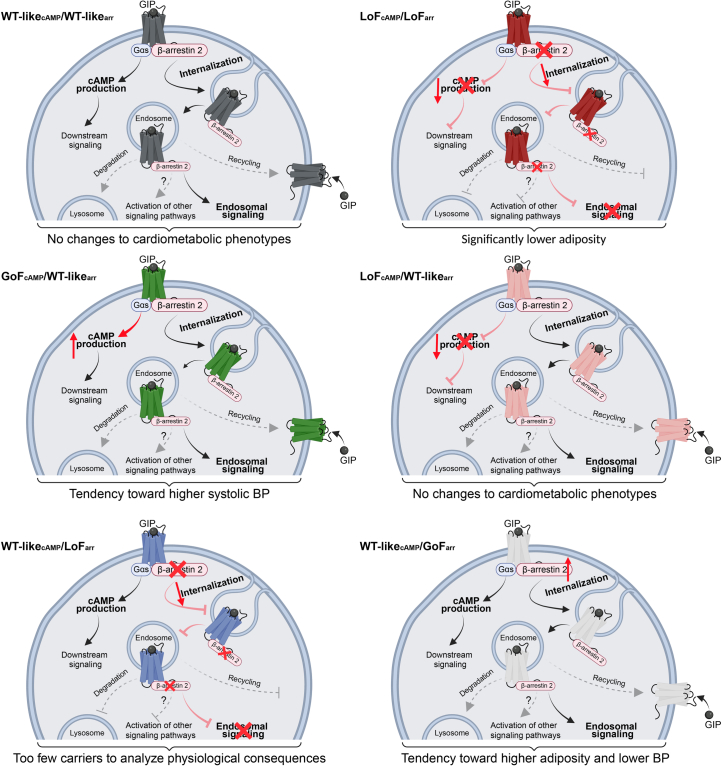
Graphical illustration of the molecular and cellular pharmacological consequences of the *GIPR* variant groups. GIP binds to the GIPR, leading to distinguishable patterns of Gαs signalling and β-arrestin recruitment, hereunder receptor internalization and endosomal signalling, depending on the GIPR variant groups. Only the LoF_cAMP_/LoF_arr_ variant group is significantly associated with lower adiposity in humans. Intracellular red arrows, including red blunted arrows, indicate which pathways are affected in the variant receptor function. A red cross indicates diminished activation of the pathway. Adapted from ‘Cellular Environment (background)’, by BioRender.com (2023). Retrieved from https://app.biorender.com/biorender-templates.

The marked clinical effects of tirzepatide may be explained by its imbalanced nature as a dual agonist with activation of GIPR as native GIP, but with less recruitment of β-arrestin to the GLP-1R with full activation of the cAMP pathway^2^. Development of similar Gs biased analogues for the GIPR has also been proposed^27^. Although it is not possible to genetically instrument the dual agonistic effects, our results and those of others^33^ highlight that decreased recruitment of β-arrestins might benefit the GLP-1R system^36,37^ but not for the GIPR regarding its role in glycaemic control. Yet our findings in the UK Biobank show beneficial effects on adiposity-associated traits of an overall reduced GIPR activation, probably due to the lost lipogenic action of GIP. Hence, our data suggest that balanced β-arrestin recruitment may drive the differences in weight-associated effects of the variants and that the contribution of β-arrestins to modulate Gs-mediated signalling of the GIPR is imperative for the biological functions of GIP (Extended Data Fig. 10).

In conclusion, we expand our current understanding of the GIP–GIPR system by highlighting the importance of β-arrestin recruitment in GIPR function. Our study points towards improving future therapeutic targeting of the GIPR system by finetuning the balance between G protein activation and β-arrestin recruitment.

## Methods

### Study populations

The Inter99 cohort is a Danish population-based cardiovascular and T2D prevention cohort initiated in 1999. All participants (30–60 years of age) underwent a comprehensive health evaluation, including measurement of anthropometrics and blood pressure, and received a 2-h 75 g oral glucose tolerance test with blood drawn throughout the test, followed by biochemical measurements. We performed targeted sequencing on 6,089 individuals from Inter99, among which 4,243 (exclusively individuals with normoglycaemia) and 5,711 individuals (without known diabetes at baseline) were available for T2D and quantitative association analyses, respectively. According to the World Health Organization 1999 criteria, 4,333 of the 5,711 individuals without diabetes at baseline had normal glucose tolerance and 1,378 had pre-diabetes. The protocol of the Inter99 study followed the Declaration of Helsinki and was approved by the local ethical committee (KA 98 155). All participants provided written consent before the study examination^38,39^.

The Danish Centre for Strategic Research in Type 2 Diabetes (DD2) cohort, initiated in 2010, is an ongoing nationwide population-based project cohort with continuous enrolment of patients with newly diagnosed T2D. At enrolment, all patients were interviewed about, for example, habitual lifestyle factors, weight gain and family history of T2D, and provided biological samples, strengthened by further linking to medical register databases with individual patient data. The DD2 study was approved by the Danish National Committee on Biomedical Research Ethics and the Danish Data Protection Agency, and all participants gave signed consent before examination^40^. We performed targeted sequencing in 2,930 individuals from this cohort. The same individuals were included in T2D association analyses.

The Holbaek Study, previously known as The Danish Childhood Obesity Biobank, is a Danish children and adolescent obesity case–control cohort initiated to manage and study childhood obesity^41^. All participants included in the current study (0.5–24.7 years of age, median age 11.7 years, *N* (>19 years) = 16, *N* (<1 year) = 1) were recruited through the Children’s Obesity Clinic (Copenhagen University Hospital Holbæk, Denmark) from January 2008 to June 2014. We performed targeted sequencing on 1,146 children or adolescents from this study cohort. The study followed the Declaration of Helsinki and was approved by the Ethics Committee of Region Zealand, Denmark (SJ-104) and the Danish Data Protection Agency. All participants provided written consent; for participants younger than 18 years, oral assent was provided by the participants and the parents provided written consent. This cohort was only used for the identification of *GIPR* variants.

The GDM cohort consists of women with diet-treated gestational diabetes between 1978 and 1985, or 1987 and 1996. The study followed the Declaration of Helsinki and was approved by the Copenhagen ethical committee (KF 11-082/01). All participants provided signed consent before examination^42^. In total, we performed targeted sequencing on 358 women from this cohort. This cohort was only used for the identification of *GIPR* variants.

The UK Biobank is a large prospective study cohort consisting of ~500,000 individuals (of mostly European ancestry) with in-depth genetic and biochemistry as well as health and lifestyle data. All participants (40–69 years of age) were recruited from across the United Kingdom in 2006–2010. The UK Biobank study was approved by the North West Centre for Research Ethics Committee (11/NW/0382), and all participants provided signed consent for health-related research^43^.

### Targeted sequencing of *GIPR* in the Danish cohorts and variant annotation

We sequenced the coding regions of 265 selected genes involved in obesity and diabetes development, including *GIPR*, by using solution-based target region capture followed by next-generation sequencing in 10,523 individuals from all four Danish study cohorts to increase the likelihood of identifying every Danish *GIPR* variant. Extensive details on DNA extraction, capture of the targeted region and next-generation sequencing were as previously described^44^. Genomic DNA extracted from peripheral blood lymphocytes was amplified by polymerase chain reaction and the final captured DNA libraries were sequenced with the Illumina HiSeq2000 platform. With the Burrows–Wheeler Alignment Tool, we aligned the final reads to the reference human genome, GRCh37/hg19 (University of California, Santa Cruz Genome Browser), then identified the genetic variants with the Genome Analysis Toolkit^45^. Individual genotypes with genotype quality <20, read depth <20, allelic depth ratio <0.2 or allelic depth ratio with a binomial test *P* value <5 × 10^−7^ were set to missing. Variants with call rate <80%, mean depth <20 and Hardy–Weinberg *P* value <5 × 10^−7^ in the subset of Inter99 individuals were removed. Individuals with mean depth <20, call rate <80%, outlying heterozygosity, non-European ancestry, more than one-degree relatives, genotype sex differing from registered sex and not matching previous genotype data were excluded.

For *GIPR*, we reached a minimum per-base mean depth of 27× and a median per-base mean coverage for the target region of 160×. We extracted variants for annotation according to the exon locations (https://genome.ucsc.edu/cgi-bin/hgTables) with 50 base pair overhangs of *GIPR* (NM_000164.3, build 37)^46,47^. Before annotation, we used LiftOver to lift the Danish dataset from GRCh37 to GRCh38, then filtered out the intronic regions and the 3′ and 5′ untranslated regions. These steps left 12 synonymous variants, two splice variants and 47 non-synonymous variants (two nonsense mutations (premature stop gain), four frameshift mutations and 41 missense mutations (Supplementary Table 1)) available for further analysis. All but the missense variant, E354Q, were rare variants (minor allele frequency (MAF) of <0.01).

### Materials

The human *GIPR* was inserted into the pcDNA 3.1 vector (GenBank accession number: NM_000164) and was synthesized by and purchased from Genscript along with the 47 non-synonymous *GIPR* variants (Supplementary Table 4). Human GIP(1–42) was purchased from Wuxi. HEK293 cells were purchased from American Type Culture Collection. Cell medium for HEK293 was purchased from Thermo Fisher Scientific. Other chemicals were purchased from standard commercial sources.

### Transfection and tissue culture

HEK293 (CRL-1573, American Type Culture Collection) and β-arrestin 1/2 knockout cells, generated from HEK293A^48^, were used for cAMP measurement, homologues competition binding and β-arrestin 2 recruitment. For the internalization assay, endosomal signalling and basal receptor localization experiments, HEK293A (R70507, Thermo Fisher) cells were used. Both cell lines were cultured at 5% CO_2_ at 37 °C in Roswell Park Memorial Institute GlutaMAX-I supplemented with 10% fetal bovine serum, 180 units ml^−1^ penicillin, and 45 g ml^−1^ streptomycin. For the cAMP and binding experiments, cells were transfected with the calcium phosphate precipitation method^49^. The polyethyleneimine (PEI) transfection method was used for the β-arrestin 2 recruitment assays^50^, endosomal signalling and basal receptor localization assays, while the lipofectamine transfection method was used for the internalization assay.

### cAMP accumulation assay

cAMP production was measured with the DiscoverX Hithunter cAMP assay according to the manufacturer’s protocol (DiscoverX). One day before the assay, the transiently transfected HEK293 cells expressing either WT GIPR or the 47 *GIPR* variants were seeded into white 96-well plates at a density of 35,000 cells per well. On the day of the assay, the cells were washed twice with HEPES-buffered saline (HBS) and incubated with HBS and 1 mM 3-isobutyl-1-methylxanthine for 30 min at 37 °C. Subsequently, the cells were stimulated with increasing concentrations of GIP(1–42) from 1 pM to 100 nM and incubated for 30 min at 37 °C. After the incubation, the assay medium was removed from the plate and the cells were washed with 30 µl PBS and treated with 40 µl enzyme donor–lysis buffer–chemiluminescent reagents and 10 µl cAMP antibodies for 60 min before 40 µl enzyme acceptor solution added. After a 3-h incubation on a shaking table in the dark, the cAMP accumulation was measured based on luminescence with a Perkin Elmer EnVision 2104 Multilabel Microplate Reader.

### Homologue competition binding assay

Binding experiments were performed simultaneously with the same transiently transfected HEK293 cells used for the cAMP experiments to enable the correlation of receptor expression with signalling between assays. In brief, 1 day before the assay, the transfected cells were seeded in a clear 96-well plate. The number of cells per well was adjusted to achieve 5–10% specific binding of ^125^I-GIP(1–42). On the assay day, the cells were washed twice with 4 °C binding buffer (50 mM HEPES buffer (pH 7.2), 1 mM CaCl_2_, 5 mM MgCl_2_ and 0.5% (wt/vol) bovine serum albumin) and incubated for 15 minutes at 4 °C. After the addition of increasing concentrations from 1 pM to 1 µM of unlabelled GIP(1–42), 15–40 pM ^125^I-GIP(1–42) was added and the plate was incubated for 3 h at 4 °C. After incubation, the cells were washed in ice-cold binding buffer and lysed with 200 mmol l^−1^ NaOH with 1% sodium dodecyl sulphate for 30 min. The gamma radiation was measured with a 2470 Wizard2 Automatic Gamma Counter.

### β-arrestin 2 recruitment assay

Measurement of β-arrestin 2 recruitment was performed with a BRET assay. Two days before the assay, the HEK293 cells were transiently transfected with either WT GIPR or the *GIPR* variants together with the donor Rluc8-Arrestin-3-Sp1, the acceptor mem-linker-citrine-SH3 and the GPCR kinase 2 to facilitate β-arrestin 2 recruitment. On assay day, the cells were washed with PBS and resuspended in PBS supplemented with 5 mmol l^−1^ glucose. Subsequently, 85 µl of the cell suspension was transferred to a white 96-well plate and 10 µl of 50 µM coelenterazine-h was added. After 10 min of incubation, increasing concentrations of GIP(1–42), from 1 pM to 1 µM, were added, the cells were incubated for 30 min at room temperature and luminescence (ratio of 535 nm over 480 nm emission) was measured with a Perkin Elmer EnVision 2104 Multilabel Microplate Reader.

### Internalization assay

The time-resolved fluorescence resonance energy transfer-based internalization assay was performed with HEK293A cells expressing the various *GIPR* variants with an N-terminal SNAP tag. The cells were seeded in white 384-well plates the day after transfection with 20,000 cells per well. The medium was removed the following day, and the variants were labelled with the Tag-lite snap-lumi4-tb (donor) (0.1 pmol µl^−1^) in Opti-MEM for 60 min at 37 °C. The cells were washed four times with internalization buffer (Hanks’ balanced salt solution (HBSS) supplemented with 1 mmol l^−1^ CaCl_2_, 1 mmol l^−1^ MgCl_2_, 20 mmol l^−1^ HEPES and 0.1% bovine serum albumin (pH 7.4)). Subsequently, 10 μl of 50 μmol l^−1^ fluorescein-*O*′-acetic acid (acceptor) was added to each well except for the wells used to record the donor signal. We then added 10 μl of GIPR agonist, in doses ranging from 1 nM to 1 µM, in the internalization buffer to the plates to monitor agonist-induced internalization. The internalization was measured every 4 min at 37 °C in a Perkin Elmer Envision 2105 multilabel reader.

### Endosomal signalling assay

HEK293A cells (350,000 per ml) were transfected in suspension with plasmid DNA expressing WT or *GIPR* variants, Rluc8-mGs, rGFP-FYVE and salmon sperm DNA (SSD) at a ratio of 2:1:6:11 that had been complexed with PEI (3:1 PEI:DNA ratio) and seeded (35,000 cells per well) in white 96-well plates pre-coated with poly-d-lysine. At 48 h post-transfection, cells were washed with HBSS and subsequently maintained in 80 µl HBSS. Cells were stimulated with 10 µl GIP(1–42) (100 pM to 1 µM) for 115 min, followed by a 5 min incubation with 10 µl NanoGlo Luciferase Assay Substrate (1:1,000, Promega). Light emission at 515/20 nm (acceptor; rGFP) and 400/70 nm (donor; Rluc8) was measured using a Tecan Spark multimode microplate reader (Männedorf).

### Basal receptor localization assay

HEK293A cells (350,000 per ml) were transfected in suspension with plasmid DNA expressing Rluc8-tagged WT or *GIPR* variants, compartment-specific acceptor (rGFP-CAAX, rGFP-FYVE, tdrGFP-Giantin or tdrGFP-PTP1B) and salmon sperm DNA at a ratio of 1:6:13 that had been complexed with PEI (3:1 PEI:DNA ratio) and seeded (35,000 cells per well) in white 96-well plates pre-coated with poly-d-lysine. At 48 h post-transfection, cells were washed with HBSS and subsequently maintained in 90 µl HBSS. Cells were incubated for 5 min with 10 µl coelenterazine 400a (2.5 µM). Light emission at 515/20 nm (acceptor; rGFP) and 400/70 nm (donor; Rluc8) was measured using a Tecan Spark multimode microplate reader (Männedorf).

### In vitro data analysis

To obtain *E*_max_, EC_50_, *B*_max_, *K*_d_ and IC_50_ values, we analysed the data with the non-linear regression curve fitting program GraphPad Prism Software 9 (GraphPad). For cAMP accumulation, the data were fitted as a sigmoidal curve with a Hill slope of 1; for homologous competition binding, the data were fitted as a sigmoidal curve with a Hill slope of −1.

The *B*_max_ was calculated according to the following equation^51^:

$${{B}}_{\max }=\frac{{{B}}_{0}\times {\text{IC}}_{50}}{[{L}]}$$

in which *B*_0_ represents the total specific binding and [*L*] is the ligand concentration. The equilibrium dissociation constant (*K*_d_) was obtained with the equation^51^:

$${{K}}_{\text{d}}={\text{IC}}_{50}-[{L}]$$

The area under the curve for *GIPR* variant internalization was calculated based on the trapezoid rule with GraphPad Prism 9.

The statistical analyses for the in vitro experiments were performed in GraphPad Prism. The order of the molecular testing of the variants was assigned randomly. Data collection and analysis were not performed blind to the conditions of the experiments.

### Variant mapping and effect predictions

All variants were subjected to Ensembl’s VEP v108 (ref. ^47^), including all dbNSFP pathogenicity predictions with rank-normalized scores (higher scores indicate higher deleteriousness), which were clustered with ‘seaborn’ clustermap^52^. Individual predictions for frameshift and stop-gained mutations including Q400Gfs*56, C84Pfs*70, E461* and W233* could not be retrieved. We aggregated individual VEPs into masking groups^15^ to categorize variants (transcript id: ENST00000590918) into putative loss-of-signalling variants, defined by passing one of the following masks: (1) LOFTEE = HC; (2) VEST4 >0.9, CADD >0.9, DANN >0.9, Eigen-raw >0.9 and Eigen-PC-raw >0.9; (3) FATHMM pred = deleterious (D), FATHMM-MKL pred = deleterious (D), PROVEAN pred = deleterious (D), MetaSVM pred = deleterious (D), MetaLR pred = deleterious (D) and MCAP >0.025; or (4) PolyPhen HDIV pred = probably damaging (D), PolyPhen HVAR pred = probably damaging (D), SIFT 4 G pred = deleterious (D), LRT pred = deleterious (D), MutTaster pred = disease_causing (D) and disease_causing_automatic (A). We excluded the PROVEAN VEP from mask 3 because of its inapplicability to evaluate variants in the GIPR transcript (ENST00000590918). We made the estimation plot with Dabestr^53^ and used the R software (v.4.0.2)^54^ to visualize the four masks and the putative loss-of-signalling variants.

We captured the *GIPR* variants in both the Danish population (combining DD2 and Inter99) and the UK Biobank that we were confident would lead to a disruption of the protein by classifying variants that had CADD scores^55^ above 30 or were protein-truncating variants (resulting in a stop gain, a start loss, a splice donor site, a splice acceptor site or a frameshift) as pLoF variants. The pLoF group in the Danish population and the UK Biobank thus consisted of nine (Supplementary Table 1) and 103 *GIPR* variants (Supplementary Table 2), respectively.

### Power calculations

No statistical methods were used to pre-determine sample sizes for the association analyses as we used fixed populations, but our sample sizes are similar to or higher than those reported in previous publications^14,34,56^.

### Statistical analyses in the Danish population

We focused on cardiometabolic phenotypes in adults, leaving 8,641 individuals for association studies of Danish adults from DD2 (ref. ^40^) and Inter99 (refs. ^38,39^). To test for T2D associations, we used DD2 as T2D cases (*N* = 2,930) and Inter99 as controls (individuals with normoglycaemia at baseline based on fasting and 2-h oral glucose tolerance test plasma glucose values (World Health Organization 1999 criteria) and without diabetes at baseline and at register-based follow-up (until 2017); *N* = 4,234), yielding a total sample size of 7,173 individuals (men/women ratio of 1.17 and average 51 years old). For quantitative trait analyses, we used those Inter99 individuals without known diabetes at baseline (*N* = 5,711, men/women ratio of 1.02 and average 46 years old). We calculated the following surrogate indices to analyse beta cell function and insulin sensitivity: the beta cell function insulin sensitivity glucose tolerance test (BIGTT)–acute insulin response (the BIGTT–AIR estimate of insulin secretion)^57^; BIGTT–sensitivity index (the BIGTT–SI estimate of insulin sensitivity); insulinogenic index (s-insulin_30 min_ – s-insulin_0 min_) / p-glucose_30 min_ (estimate of insulin secretion) and the insulin sensitivity index Matsuda (estimate of insulin sensitivity)^58^.

We used R (v.4.0.2)^54^ to perform logistic regression for T2D risk analyses, linear regression for analysis of quantitative traits and burden tests for all phenotypes. For the burden tests (using equal weights for all variants), we used the R package ‘SKAT’ (v.2.2.5, https://cran.r-project.org/web/packages/SKAT/SKAT.pdf)^59^ and applied the SKAT burden test (‘r.corr = 1’) for variants tested in vitro and SKAT-O (‘method = ‘optimal.adj’’) for synonymous and pLoF variants^60^. We classified carriers as individuals carrying any of the *GIPR* variants, according to their in vitro result grouping. We rank-normalized all quantitative traits before analysis for data to have normal distribution, and we adjusted for sex, age, age^2^ and four principal components (PCs), as well as BMI where mentioned. We imputed missing genotype data but excluded individuals with missing phenotypes or covariates. We only included variant groups with more than five carriers to have acceptable representatives of carriers, excluding the GoF_cAMP_/WT-like_arr_ and WT-like_cAMP_/GoF_arr_ groups for burden testing. We did not perform sex-stratified analyses because of the limited number of carriers in the variant groups, which may further limit the statistical power. However, we accounted for sex-related effects by using sex (reported at recruitment) as a covariate in our statistical analyses. We controlled the false discovery rate (FDR) with p.adjust in R (v.4.0.2)^54^ to calculate Benjamini–Hochberg *q*-values^61^.

### Statistical analyses in the UK Biobank

The UK Biobank 470K whole-exome sequencing pVCF file^18^ was filtered to samples passing the following filters: not outliers for heterozygosity and missing rate (field #22027), no sex chromosome aneuploidy (#22019), not of mixed ethnic background (#21000), call rate >0.9 and no withdrawn consent. We further removed single-nucleotide variation genotypes with sequencing depth (DP) <7 and indels with DP <10, entries with a genotype quality <20, homozygous reference call with allele balance (AB) >0.1, heterozygous alternate with AB <0.9 and heterozygous with AB not in (0.2, 0.9) (ref. ^62^). Variants were restricted to those in whole-exome sequencing capture regions, with more than 90% of genotypes having DP >10. Variants with a call rate <0.99 and Hardy–Weinberg equilibrium *P* < 10 × 10^−15^ were removed. We annotated the variants with VEP^47^. All phenotypes, including BMI (#21001), body fat percentage (#23099), waist circumference (#48), systolic blood pressure (#93), diastolic blood pressure (#94), obesity/E66 (#130792), hypertension/I10 (#131286) and non-insulin-dependent (type II) diabetes mellitus/E11 (#130708) were pre-processed with PHESANT^63^ and inverse rank normal transformed for data to have normal distribution. We used the ICD-10 code for obesity (E66) to provide a more nuanced picture of the body composition as BMI represents a single snapshot of an individual; in particular, 14,380 samples had an E66 diagnosis and BMI <30; 38,427 samples had an E66 diagnosis and BMI >30; 79,013 samples had no E66 diagnosis, but BMI >30; and 282 samples had neither E66 diagnosis nor a BMI measure. We opted not to filter related individuals, since Regenie is robust against percentages of related individuals as high as 50%^64^ and this cohort exhibited a percentage of approximately 22%^65^. In total, 465,506 samples (54% were women, average 57 years old and 91% of British ancestry) were available for analysis after quality control filtering and were not filtered further for ancestry. The 427,931 United Kingdom individuals were defined by Euclidean distance on principal component analysis space^66^. Variant quality control metrics are provided in Supplementary Table 2. All quality control was performed with Hail (Hail Team. Hail 0.2.64 https://github.com/hail-is/hail).

Multiple variant combination association tests were performed with Regenie 3.2.5 (ref. ^65^) adjusting for sex, age, age^2^, age × sex, age^2^ × sex and the first 20 ancestry-informative PCs. All analyses were performed on the UK Biobank Research Analysis Platform Spark clusters (Hail) or single nodes (Regenie). The Cauchy *P* value combination test was performed with the omnibus aggregated Cauchy association test (ACAT-O)^67^. Regenie step 1 was run according to UK Biobank recommendations (https://rgcgithub.github.io/regenie/recommendations/), and exact step two parameters are available in the GitHub repo scripts folder. Per tested phenotype association, individuals with missing values in either the phenotype, covariates or genotypes were excluded from the analysis. Benjamini–Hochberg *q*-values^61^ were calculated with p.adjust in R (v.4.0.2)^54^ to adjust for multiple comparisons. We did not perform sex-stratified analyses because of the limited number of carriers in the variant groups, which may further limit the statistical power. However, we accounted for sex-related effects by using sex (derived from the National Health Service or self-reported) as a covariate in our statistical analyses.

To create the forest plots of the association analyses from the Danish population and the UK Biobank, we used the R package ‘metafor’ (v.4.4.0, https://cran.r-project.org/web/packages/metafor/metafor.pdf)^68^.

For the meta-analysis regression, phenotypic estimates of single variants with an allele count >20 were obtained, leaving 94 variants for analysis, of which 17 were in vitro-tested variants. These estimates derived from the association tests using Regenie^65^. Single-variant estimates of phenotypes and in vitro cAMP production and β-arrestin recruitment were modelled in Bayesian regression models using Stan (BRMS)^69^ in R (v.4.3.0)^54^ as ‘phenotype | mi(SE) ~ mi(cAMP) * mi(Barr)’ using the approach described in the vignette ‘Handle Missing Values with brms’. We used normal(0,1) priors for effect sizes and exponential(1) priors for sigma. We used four chains with 10,000 iterations with 4,000 warmups. All chains converged with a potential scale reduction factor onsplit chains (Rhat) 1.00 or 1.01 and a satisfactory effective sample size. Each phenotype was modelled separately, and fixed effects were plotted based on 1,000 samples. See ‘Code availability’ for exact model parameters.

### Pharmacologic studies in *Arrb2* KO mice

Studies were approved by and performed according to the guidelines of the Institutional Animal Care and Use Committee of Duke University. Male *Arrb2* KO mice (Arrb2^−/−^) ~12–15 weeks of age were generously provided by Drs Howard Rockman and Robert Lefkowitz at Duke University^70^. The mice were kept in standard housing conditions with a temperature of 20–26 °C and a 12-h dark/light cycle to ensure the physiological and psychological wellbeing of the mice. Mice were fed research diets 5053. We restrictedly used male mice because of the potential impact of the sexual dimorphism, that is, the oestrus cycle in female mice on cardiometabolic traits, resulting in unwanted variability and to ensure comparability to existing research predominantly using male mice^71^. The mice were subjected to an IPGTT. Test compounds, including native GLP-1 and d-Ala GIP (GIP(1–42) with a substitution of Ala with d-Ala at position 2), were injected 15 min before glucose in these experiments. Data collection and analysis were not randomized or blind to the conditions of the experiments. The statistical analysis was performed in GraphPad Prism.

### Reporting summary

Further information on research design is available in the Nature Portfolio Reporting Summary linked to this article.

### Supplementary information

Supplementary Information Supplementary Figs. 1–3.

Reporting Summary

Supplementary Table 1 Discovery of Danish protein-coding *GIPR* variants. A list of the 59 protein-coding *GIPR* variations identified through targeted sequencing in four Danish study cohorts: HOLBAEK Study, GDM, DD2 and Inter99. AA change, amino acid change; alt allele, alternative allele; ref allele, reference allele; SNV, single-nucleotide variation; *N*, total number of individuals within the cohort; hetero/homo, heterozygous carriers of the alternative allele/homozygous carriers of the alternative allele; MAF, minor allele frequency.

Supplementary Table 2 Tab ‘All UKBB samples’: Supplementary Table 2. Discovery of protein-coding *GIPR* variants in the UK Biobank and the overlap with the in vitro-tested *GIPR* variants. A list of the protein-coding *GIPR* variants identified in the UK Biobank for all samples. The overlapping non-synonymous *GIPR* variants are shown in ‘Grouping’, denoted by the *GIPR* variant groups, as are the synonymous and pLoF variants. Abbreviations and column explanation: locus, chromosome and position; ref, reference allele; alt, alternate allele; VEP, variant effect predictor; CADD, combined annotation dependent depletion score; *N*, total sample size; *N* not called, samples excluded; *N*_QC, samples excluded during QC; heterozygous alt, number of heterozygous carriers of the alternate allele; *N* homozygous (ref, alt), number of individuals being homozygous carriers of the reference allele and alternate allele, respectively; total alt, total number of carriers of the alternate allele; *P* value HWE, *P* value for Hardy–Weinberg equilibrium (HWE), derived from a two-sided exact test with mid-*P* value correction of HWE via the Levene–Haldane distribution; alleles, the reference and alternate allele; NA, not applicable. Tab ‘UK ancestry samples only’: Supplementary Table 2. Discovery of protein-coding *GIPR* variants in the UK Biobank and the overlap with the laboratory-tested *GIPR* variants. A list of the protein-coding *GIPR* variants identified in the UK Biobank of individuals with United Kingdom ancestry. The overlapping nonsynonymous *GIPR* variants are shown in ‘Grouping’, denoted by the GIPR variant groups, as are the synonymous and pLoF variants. Abbreviations and column explanation: locus, chromosome and position; ref, reference allele; alt, alternate allele; VEP, variant effect predictor; CADD, combined annotation dependent depletion score; *N*, total sample size; *N* not called, samples excluded; *N*_QC, samples excluded during QC; heterozygous alt, number of heterozygous carriers of the alternate allele; *N* homozygous (ref, alt), number of individuals being homozygous carriers of the reference allele and alternate allele, respectively; total alt, total number of carriers of the alternate allele; *P* value HWE, *P* value for Hardy–Weinberg equilibrium (HWE), derived from a two-sided exact test with mid-*P* value correction of HWE via the Levene–Haldane distribution; alleles, the reference and alternate allele; NA, not applicable.

Supplementary Table 3 Summary statistics of the T2D association studies in the Danish population. s.d., standard deviation; *P*-glm, *P* value of a general linear model (logistic regression); *P*, *P* value of the burden test (performed using SKAT/SKAT-O); *q*, false discovery rate (FDR) adjusted *P*.

Supplementary Table 4 Sequence of *GIPR* variants. The nucleotide changes were introduced into the human *GIPR* (GenBank accession number: NM_000164) in pcDNA 3.1 vector (sequence not provided here). The site-directed mutagenesis was performed by Genscript (Piscataway, NJ).

### Source data

Source Data Fig. 1 cAMP and binding in vitro source data.

Source Data Fig. 2 β-arrestin recruitment in vitro source data.

Source Data Fig. 3 Statistical UK Biobank source data.

Source Data Fig. 4 Endosomal signalling and in vivo source data.

Source Data Extended Data Fig. 1/Table 1 cAMP and binding in vitro source data.

Source Data Extended Data Fig. 2/Table 2 Variant effect predictor source data.

Source Data Extended Data Fig. 3/Table 3 Internalization in vitro source data.

Source Data Extended Data Fig. 4/Table 4 Statistical Danish cohorts source data.

Source Data Extended Data Fig. 5/Table 5 Statistical Danish cohorts source data.

Source Data Extended Data Fig. 6/Table 6 Statistical UK Biobank source data.

Source Data Extended Data Fig. 7/Table 7 Statistical UK Biobank source data.

Source Data Extended Data Fig. 8/Table 8 Single-variant UK Biobank source data.

Source Data Extended Data Fig. 9/Table 9 In vitro source data.

## Data Availability

The reference human genome GRCh37/hg19 is available at the University of California, Santa Cruz Genome Browser (https://genome.ucsc.edu/). Data from the Danish cohorts can only become available from corresponding author N.G. upon reasonable request due to handling of personal information. For requesting the DD2 data, submission of an application (https://dd2.dk/forskning/ansoeg-om-data) must be directed to Research Manager Kurt Højlund (Kurt.Hoejlund.rsyd.dk) and Program Manager Jens S. Nielsen (jsn@rsyd.dk). The application form for accessing the UK Biobank data sets can be found at https://www.ukbiobank.ac.uk/enable-your-research. Source data are provided with this paper.

## Code availability

Code used for the analyses of the Danish cohorts can become available from corresponding author Niels Grarup upon request due to handling of personal information. All code used for the analyses of the UK Biobank data is available at https://github.com/HauserGroup/gogoGPCR.
