# Supplementary material for: Characterization of genetic variants of GIPR reveals a contribution of β-arrestin to metabolic phenotypes
Source: Nat Metab. 2024 Jun 13;6(7):1268–81. doi: 10.1038/s42255-024-01061-4 (PMC11272584; doi:10.1038/s42255-024-01061-4)
Supplement: Supplementary file 1 — Supplementary Figs. 1–3. [file 42255_2024_1061_MOESM1_ESM.pdf]

nature metabolism

Supplementary information https://doi.org/10.1038/s42255-024-01061-4

Characterization of genetic variants of *GIPR* reveals a contribution of β-arrestin to metabolic phenotypes

In the format provided by the authors and unedited

Supplementary Fig. 1

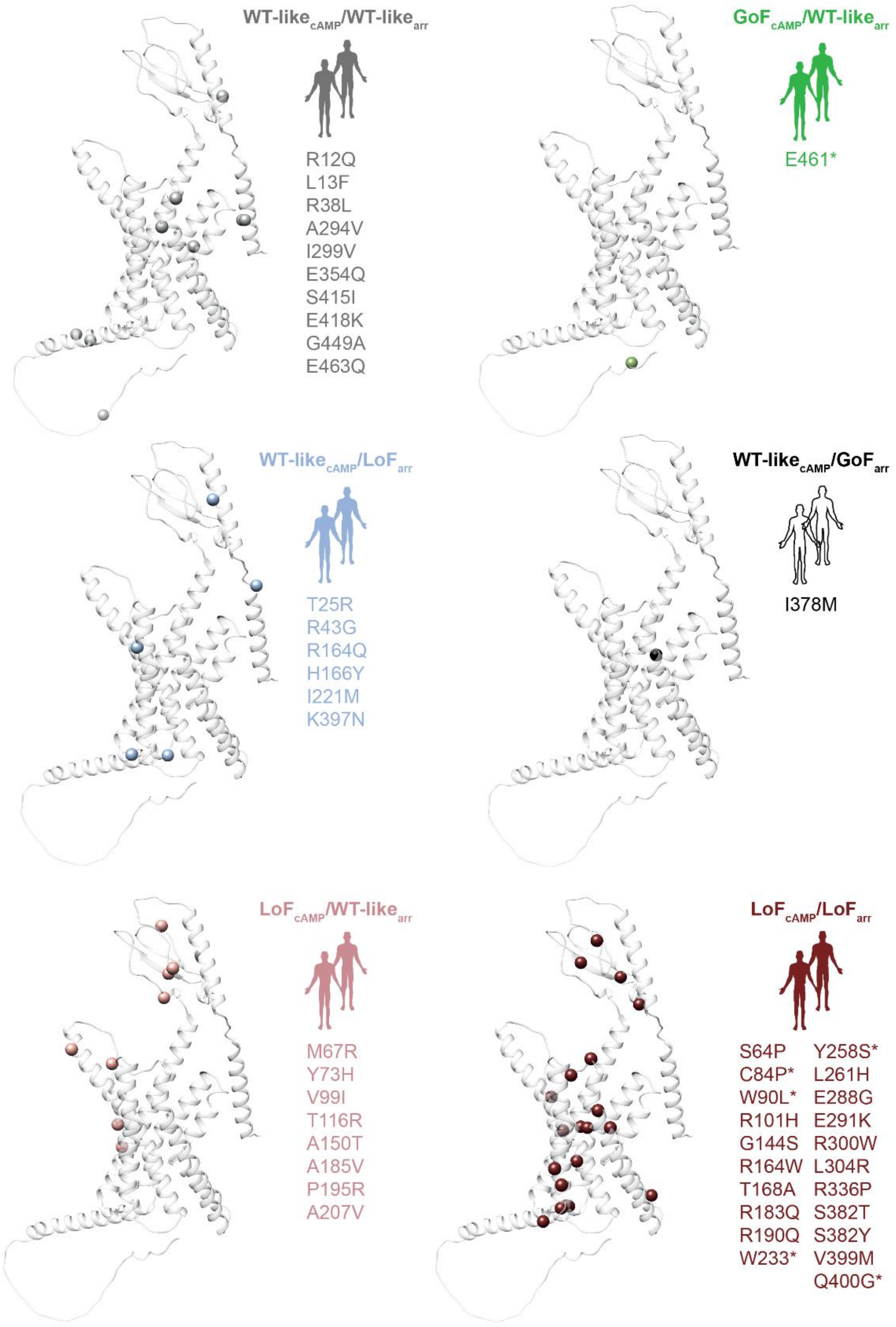

Supplementary Fig. 1. Structural mapping of variant groupings. Mapping of all 47 *GIPR* variants by their *in vitro*-based groupings onto an AlphaFold-generated GIPR structure.

Supplementary Fig. 2

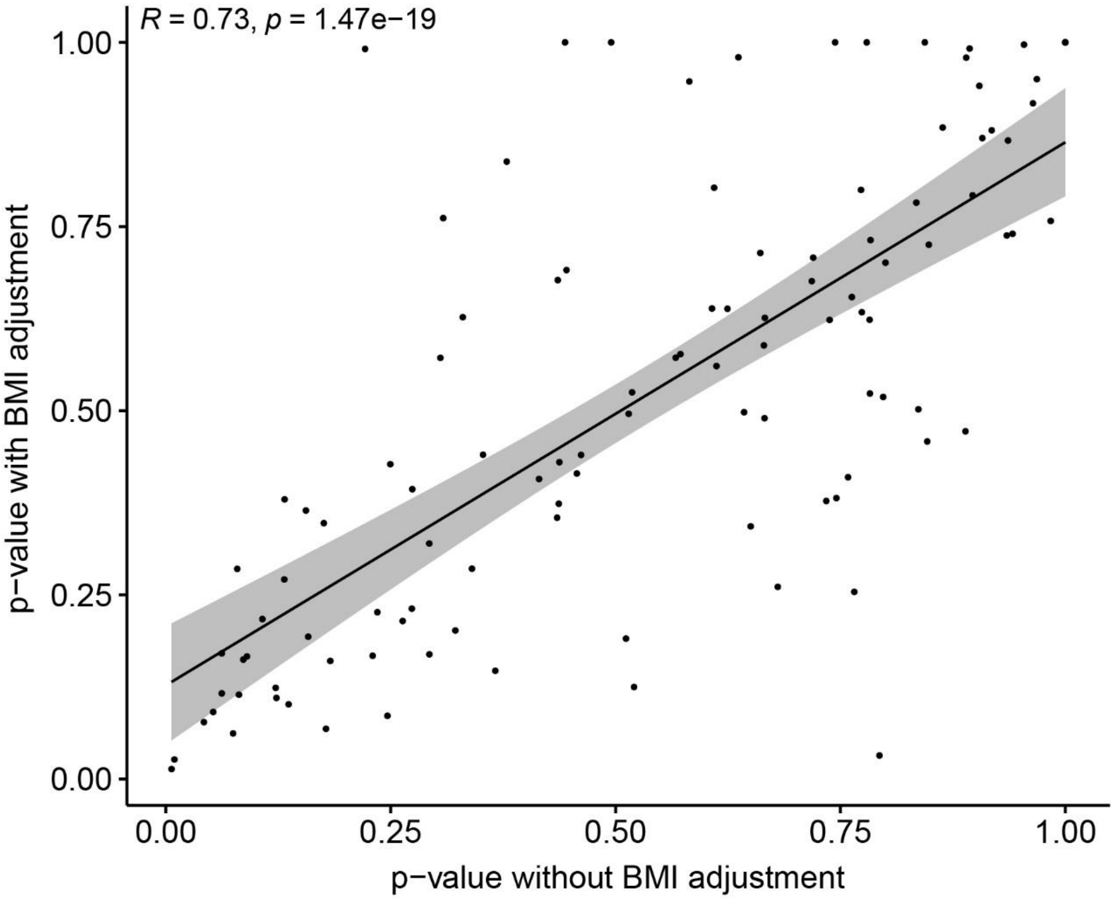

Supplementary Fig. 2. Correlation between p-values adjusted for BMI or not in the Danish population. Correlation plot of p-values for burden tests with/without body mass index (BMI) adjustment for type 2 diabetes and quantitative traits, showing the regression line and the 95% confidence interval (error band). The x-axis shows the p-values without BMI adjustment; the y-axis shows the p-values with BMI adjustment. R, Pearson's correlation coefficient; *p*, p-value of the correlation (two-sided Pearson's correlation coefficient test).

Supplementary Fig. 3

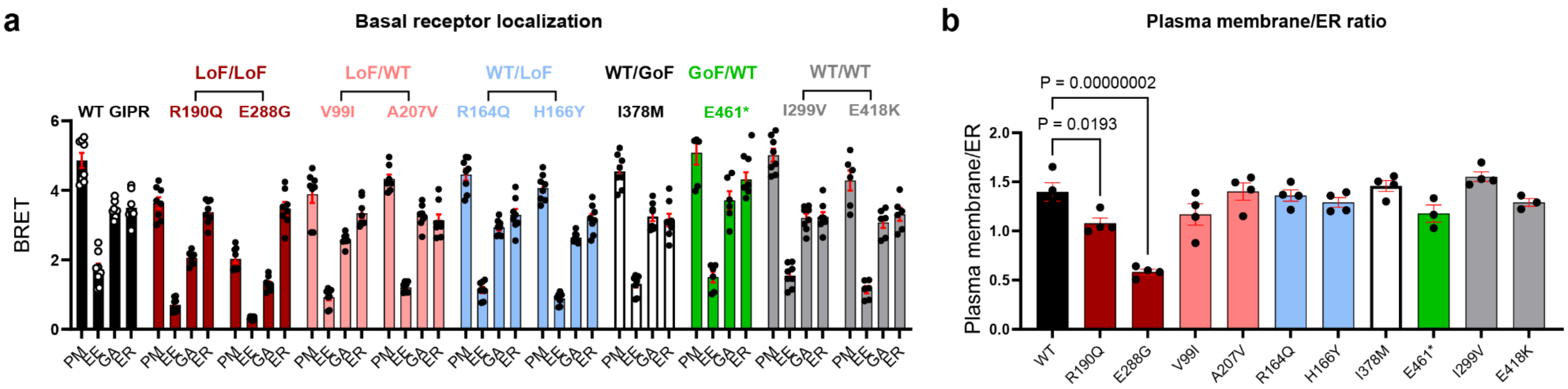

Supplementary Fig. 3. Basal subcellular localization of WT GIPR and variants. a, The distribution of receptor variants across different cellular compartments determined with compartment-specific markers: PM (plasma membrane), EE (endosomes), GA (trans-Golgi apparatus) and ER (endoplasmic reticulum). LoF/LoF (LoFcAMP/LoFarr), LoF/WT (LoFcAMP/WT-likearr), WT/LoF (WT-likecAMP/LoFarr), WT/GoF, (WT-likecAMP/GoFarr), GoF/WT (GoFcAMP/WT-likearr), WT/WT (WT-likecAMP/WT-likearr). WT GIPR, R190Q, E288G, V99I, A207V, R164Q, H166Y, I378M, I299V, N = 8. E418K, E461*, N = 6 **b**, The BRET ratio of PM versus ER localization of receptor variants. The statistical significance between R190Q and E288G with WT GIPR was determined by one-way analysis of variance (Dunnett's multiple comparisons test). WT GIPR, R190Q, E288G, V99I, A207V, R164Q, H166Y, I378M, I299V, N = 8. E418K, E461*, N = 6. N represents biologically independent experiments. Independent experiments were performed in duplicates. The data represent the mean ± SEM.
