# Supplementary material for: Characterization of genetic variants of GIPR reveals a contribution of β-arrestin to metabolic phenotypes
Source: Nat Metab. 2024 Jun 13;6(7):1268–81. doi: 10.1038/s42255-024-01061-4 (PMC11272584; doi:10.1038/s42255-024-01061-4)
Supplement: Supplementary file 2 — Reporting Summary [file 42255_2024_1061_MOESM2_ESM.pdf]

nature portfolio

Corresponding author(s): Niels Grarup, Alexander S. Hauser & Mette M. Rosenkilde

Last updated by author(s): Apr 23, 2024

Reporting Summary

Nature Portfolio wishes to improve the reproducibility of the work that we publish. This form provides structure for consistency and transparency in reporting. For further information on Nature Portfolio policies, see our Editorial Policies and the Editorial Policy Checklist.

Statistics

For all statistical analyses, confirm that the following items are present in the figure legend, table legend, main text, or Methods section.

n/a	Confirmed	
☐	☒	The exact sample size (*n*) for each experimental group/condition, given as a discrete number and unit of measurement
☐	☒	A statement on whether measurements were taken from distinct samples or whether the same sample was measured repeatedly
☐	☒	The statistical test(s) used AND whether they are one- or two-sided<br>*Only common tests should be described solely by name; describe more complex techniques in the Methods section.*
☐	☒	A description of all covariates tested
☐	☒	A description of any assumptions or corrections, such as tests of normality and adjustment for multiple comparisons
☐	☒	A full description of the statistical parameters including central tendency (e.g. means) or other basic estimates (e.g. regression coefficient) AND variation (e.g. standard deviation) or associated estimates of uncertainty (e.g. confidence intervals)
☐	☒	For null hypothesis testing, the test statistic (e.g. *F*, *t*, *r*) with confidence intervals, effect sizes, degrees of freedom and *P* value noted<br>*Give P values as exact values whenever suitable.*
☒	☐	For Bayesian analysis, information on the choice of priors and Markov chain Monte Carlo settings
☒	☐	For hierarchical and complex designs, identification of the appropriate level for tests and full reporting of outcomes
☐	☒	Estimates of effect sizes (e.g. Cohen's *d*, Pearson's *r*), indicating how they were calculated

Our web collection on statistics for biologists contains articles on many of the points above.

Software and code

Policy information about availability of computer code

Data collection	For cAMP accumulation and arrestin recruitment, PerkinElmer Envision 2104 Multilabel Microplate Reader (Waltham, Massachusetts, USA) were used. For homologue competition binding assay, 2470 Wizard2 Automatic Gamma Counter (Waltham, Massachusetts, USA) was used for measurement. For internalization assay the Perkin Elmer Envision 2015 multilabel (Waltham, Massachusetts, USA) reader was used. Endosomal and localization assay were measured with Tecan Spark multimode microplate reader (Männedorf, Switzerland).
Data analysis	Software/packages used in data analysis: GraphPad Prism 9, Regenie (v.3.2.5), Hail (v.0.2.64), R (v.4.0.2 and v.4.3.0), Ensembl's VEP v108, PHESANT (https://github.com/MRCIEU/PHESANT), ACAT-O (as implemented in Regenie), R package "SKAT" (v.2.2.5), R package "metafor" (v.4.4.0), R package "stats" for the p.adjust function (v.4.3.2), and R package "BRMS" (v.2.19).<br><br>For UK Biobank analyses, code can be found: https://github.com/HauserGroup/gogoGPCR, which is publicly available.<br><br>For the analyses in Danish cohorts, codes can be accessible from corresponding author Niels Grarup upon request.

For manuscripts utilizing custom algorithms or software that are central to the research but not yet described in published literature, software must be made available to editors and reviewers. We strongly encourage code deposition in a community repository (e.g. GitHub). See the Nature Portfolio guidelines for submitting code & software for further information.

Data

Policy information about availability of data

All manuscripts must include a data availability statement. This statement should provide the following information, where applicable:

- Accession codes, unique identifiers, or web links for publicly available datasets
- A description of any restrictions on data availability
- For clinical datasets or third party data, please ensure that the statement adheres to our policy

The reference human genome GRCh37/hg19 is available at the UCSC Genome Browser (https://genome.ucsc.edu/). Some or all data used in the current study may become available from the corresponding authors upon reasonable request. For requesting the DD2 data, submission of an application (https://dd2.dk/forskning/ansoeg-om-data) must be directed to Research Manager Kurt Højlund (Kurt.Hoejlund.rsyd.dk) and Program Manager Jens S. Nielsen (jsn@rsyd.dk). The application form for accessing the UK Biobank data sets can be found at https://www.ukbiobank.ac.uk/enable-your-research.
Source data are provided with this paper.

Research involving human participants, their data, or biological material

Policy information about studies with human participants or human data. See also policy information about sex, gender (identity/presentation), and sexual orientation and race, ethnicity and racism.

Reporting on sex and gender

Since the variants evaluated in the current study are rare, we have a limited number of carriers in both the Danish cohorts and the UK Biobank (for some groups) as can be seen in Fig. 3c and Extended Data Fig. 4 and 5. Hence, stratifying for sex may come with the cost of insufficient power, and are therefore not suitable for our study to ensure consistency between analysis in the Danish cohorts and in the UK Biobank. However, we adjust for sex-related effects in our statistical analysis in the Danish cohorts and UK Biobank

Reporting on race, ethnicity, or other socially relevant groupings

Only Danish individuals (self-reported) are used for the analyses using the Danish cohorts. For the UK Biobank analyses, we did the analyses in a sample of mixed ancestry (considering population structure by Regenie), and in a sample of individuals only with British ancestry (self-reported).

Population characteristics

The Inter99 cohort is a Danish population-based cardiovascular and T2D prevention cohort initiated in 1999. All participants (30–60 years of age) underwent a comprehensive health evaluation, including measurement of anthropometrics and blood pressure, and received a 2-hour 75 g oral glucose tolerance test (OGTT) with blood drawn throughout the test, followed by biochemical measurements. We performed targeted sequencing on 6,089 individuals from Inter99, among which 4,243 (exclusively individuals with normoglycemia) and 5,711 individuals (without known diabetes at baseline) were available for T2D and quantitative association analyses, respectively. According to the WHO 1999 criteria, 4,333 of the 5,711 individuals without diabetes at baseline had normal glucose tolerance, and 1,378 had prediabetes.

The Danish Centre for Strategic Research in Type 2 Diabetes (DD2) cohort, initiated in 2010, is an ongoing nationwide population-based project cohort with continuous enrolment of patients with newly diagnosed T2D. At enrolment, all patients were interviewed about, e.g., habitual lifestyle factors, weight gain, and family history of T2D and provided biological samples, strengthened by further linking to medical register databases with individual patient data.

The Holbaek Study, previously known as The Danish Childhood Obesity Biobank, is a Danish children/adolescent obesity case-control cohort initiated to manage and study childhood obesity. All participants included in the current study (0.5–24.7 years of age, median age = 11.7 years, N >19 years = 16, N <1 year = 1) were recruited through the Children's Obesity Clinic (Copenhagen University Hospital Holbæk, Denmark) from January 2008 to June 2014. We performed targeted sequencing on 1,146 children/adolescents from this study cohort.

The GDM cohort consists of women with diet-treated gestational diabetes between 1978 and 1985, or 1987 and 1996.

The UK Biobank is a large prospective study cohort consisting of ~500,000 individuals (of mostly European ancestry) with in-depth genetic, biochemistry as well as health and lifestyle data. All participants (40–69 years of age) were recruited from across the UK in 2006–2010.

Recruitment

We did not recruit any participants.

Ethics oversight

All human studies, from which we used data from, obtained all required approvals, and all participants provided written consent prior to participation:

The protocol of the Inter99 study followed the Declaration of Helsinki and was approved by the local ethical committee (KA 98 155). All Inter99 participants provided written consent before the study examination.

The DD2 study was approved by the Danish National Committee on Biomedical Research Ethics and the Danish Data Protection Agency, and all participants gave signed consent before examination.

The Holbaek study followed the Declaration of Helsinki and was approved by the Ethics Committee of Region Zealand, Denmark (SJ-104) and the Danish Data Protection Agency. All participants of the Holbaek study provided written consent; for participants younger than 18 years, oral assent was provided by the participants, and the parents provided written consent.

The GDM cohort study followed the Declaration of Helsinki and was approved by the Copenhagen ethical committee (KF)

11-082/01). All GDM study participants provided signed consent prior to examination.

The UK Biobank study was approved by the North West Centre for Research Ethics Committee (11/NW/0382), and all participants provided signed consent for health-related research.

Note that full information on the approval of the study protocol must also be provided in the manuscript.

Field-specific reporting

Please select the one below that is the best fit for your research. If you are not sure, read the appropriate sections before making your selection.

☒ Life sciences ☐ Behavioural & social sciences ☐ Ecological, evolutionary & environmental sciences

For a reference copy of the document with all sections, see nature.com/documents/nr-reporting-summary-flat.pdf

Life sciences study design

All studies must disclose on these points even when the disclosure is negative.

Sample size	The sample size for the in vitro experiments consisted of at least three independent experiments, a standard practice in the cell biology field to discern differences. The sample size of the mouse study was chosen based on ethical consideration (3Rs). We did not perform power calculation, because we used fixed populations, i.e., we were not able to include more individuals for the analyses of these rare variants. It maytherefore not make sense to perform a power calculations but rather to look at the specific effect sizes and confidence interval, which may be more informative about power within a given population. Even though, we have a rather small sample size in the Danish cohorts (N for targeted sequencing = 10,523, and N for association testing = up to 8,641), we use the UK Biobank, the largest cohort with whole-exome data (N = up to 465,506). These sample sizes are similar to or larger than used in previous studies [PMID: 37709961, PMID: 37235780, PMID: 31002796].
Data exclusions	No data points were excluded from the in vitro and in vivo experiments. All exclusion criteria for the sequencing data and the statistical analyses in the Danish cohorts and in the UK Biobank are described in the methods section.
Replication	The molecular pharmacological experiments were conducted at least three times, using either duplicate or triplicate technical replicates, with all replication attempts being successful.
Randomization	The order of the molecular testing of the variants was assigned randomly.
Blinding	Blinding was not conducted for the in vitro experiments due to their complexity, which rendered the process impractical. Furthermore, the use of machine-based measurements minimized potential investigator bias.

Reporting for specific materials, systems and methods

We require information from authors about some types of materials, experimental systems and methods used in many studies. Here, indicate whether each material, system or method listed is relevant to your study. If you are not sure if a list item applies to your research, read the appropriate section before selecting a response.

Materials & experimental systems

n/a	Involved in the study	
☒	☐	Antibodies
☐	☒	Eukaryotic cell lines
☒	☐	Palaeontology and archaeology
☐	☒	Animals and other organisms
☒	☐	Clinical data
☒	☐	Dual use research of concern
☒	☐	Plants

Methods

n/a	Involved in the study	
☒	☐	ChIP-seq
☒	☐	Flow cytometry
☒	☐	MRI-based neuroimaging

Eukaryotic cell lines

Policy information about cell lines and Sex and Gender in Research

Cell line source(s)	The kidney of human embryo (HEK293) was purchased from ATCC (cat. no.: CRL-1573, Manassas, Virginia). HEK293A was purchased from ThermoFisher (cat. no.: R70507, Waltham, Massachusetts). β-arrestin 1/2 knockout cells were generated from HEK293A cells by Asuka Inoue (PMID: 28634209).
Authentication	The HEK293 and HEK293A cell line was purchased from the company ATCC and ThermoFisher, respectively and authenticated by STR sequencing by the companies.
Mycoplasma contamination	Not detected. All cell lines are frequently (once a month) tested for mycoplasma in our laboratory by PCR.

Commonly misidentified lines (See ICLAC register)	We did not use any commonly misidentified cell lines.

Animals and other research organisms

Policy information about studies involving animals; ARRIVE guidelines recommended for reporting animal research, and Sex and Gender in Research

Laboratory animals	Male beta-arrestin 2 knockout mice (C57BL/6J background) aged ~12-15 weeks were generously provided by Drs Howard Rockman and Robert Lefkowitz at Duke University. The mice were kept in standard housing conditions with a temperature of 20-26 degrees and a 12-hr dark/light cycle to ensure the physiological and psychological well-being of the mice. Mice were fed research diets, 5053.
Wild animals	The study did not involve wild animals.
Reporting on sex	We only used male mice, and the reason is stated in the methods section.
Field-collected samples	The study did not involve samples collected from the field.
Ethics oversight	Research carried out for this study was handled according to approved protocols and animal welfare regulation of Duke University Medical Center's institutional Review Boards: The studies were approved by and performed according to the guidelines of the Institutional Animal Care and Use Committee of Duke University

Note that full information on the approval of the study protocol must also be provided in the manuscript.
